# PGF_2α_ induces a pro-labour phenotypical switch in human myometrial cells that can be inhibited with PGF_2α_ receptor antagonists

**DOI:** 10.3389/fphar.2023.1285779

**Published:** 2023-12-14

**Authors:** Isabel Hamshaw, Anne Straube, Richard Stark, Laura Baxter, Mohammad T. Alam, Walter J. Wever, Jun Yin, Yong Yue, Philippe Pinton, Aritro Sen, Gregory D. Ferguson, Andrew M. Blanks

**Affiliations:** ^1^ Clinical Science Research Laboratories, Division of Biomedical Sciences, Warwick Medical School, University of Warwick, Coventry, United Kingdom; ^2^ Centre for Mechanochemical Cell Biology, Division of Biomedical Sciences, University of Warwick, Coventry, United Kingdom; ^3^ Bioinformatics RTP, Warwick Medical School, University of Warwick, Coventry, United Kingdom; ^4^ Ferring Research Institute Inc., San Diego, United Kingdom; ^5^ Ferring Pharmaceuticals, International PharmaScience Center, Kastrup, Denmark

**Keywords:** myometrium, PGF2α, FP receptor, FP antagonists, smooth muscle, labour, preterm birth

## Abstract

Preterm birth is the leading cause of infant morbidity and mortality. There has been an interest in developing prostaglandin F_2α_ (PGF_2α_) antagonists as a new treatment for preterm birth, although much of the rationale for their use is based on studies in rodents where PGF_2α_ initiates labour by regressing the corpus luteum and reducing systemic progesterone concentrations. How PGF_2α_ antagonism would act in humans who do not have a fall in systemic progesterone remains unclear. One possibility, in addition to an acute stimulation of contractions, is a direct alteration of the myometrial smooth muscle cell state towards a pro-labour phenotype. In this study, we developed an immortalised myometrial cell line, MYLA, derived from myometrial tissue obtained from a pregnant, non-labouring patient, as well as a novel class of PGF_2α_ receptor (FP) antagonist. We verified the functionality of the cell line by stimulation with PGF_2α_, resulting in Gα_q_-specific coupling and Ca^2+^ release, which were inhibited by FP antagonism. Compared to four published FP receptor antagonists, the novel FP antagonist N582707 was the most potent compound [F_max_ 7.67 ± 0.63 (IC_50_ 21.26 nM), AUC 7.30 ± 0.32 (IC_50_ 50.43 nM), and frequency of Ca^2+^ oscillations 7.66 ± 0.41 (IC_50_ 22.15 nM)]. RNA-sequencing of the MYLA cell line at 1, 3, 6, 12, 24, and 48 h post PGF_2α_ treatment revealed a transforming phenotype from a fibroblastic to smooth muscle mRNA profile. PGF_2α_ treatment increased the expression of *MYLK*, *CALD1*, and *CNN1* as well as the pro-labour genes *OXTR*, *IL6*, and *IL11*, which were inhibited by FP antagonism. Concomitant with the inhibition of a smooth muscle, pro-labour transition, FP antagonism increased the expression of the fibroblast marker genes *DCN*, *FBLN1*, and *PDGFRA*. Our findings suggest that in addition to the well-described acute contractile effect, PGF_2α_ transforms myometrial smooth muscle cells from a myofibroblast to a smooth muscle, pro-labour–like state and that the novel compound N582707 has the potential for prophylactic use in preterm labour management beyond its use as an acute tocolytic drug.

## Introduction

Every year, 14.9 million babies are born preterm (<37 weeks gestation), representing 11.1% of live births (Chawanpaiboon et al., 2019). Preterm birth (PTB) is the leading global cause of infant morbidity and mortality, accounting for 18% of all deaths in children aged under 5 years old (Goldenberg et al., 2008; Mangham et al., 2009; Walani, 2020). The current treatment for treating life-threatening PTB is tocolytics, which include the calcium channel blocker, nifedipine, and the oxytocin receptor antagonist, atosiban (Vogel et al., 2014; Lamont and Jørgensen, 2019). Whilst these tocolytics can delay labour for ≤48 h, they do not prevent PTB and have shown limited improvement in both short-term and long-term neonatal outcomes (Schwarz and Page, 2003; Vogel et al., 2014; Lamont and Jørgensen, 2019).

The prostaglandin F2α (FP) receptor is a G protein–coupled receptor (GPCR) that is expressed in the human eye and myometrium and is upregulated during inflammation (Matsumoto et al., 1997; Mukhopadhyay et al., 2001; Beck et al., 2020) It is well established that prostaglandins play a dominant role during human parturition (Casey and MacDonald, 1988; Senior et al., 1993; Mitchell et al., 1995; Brodt-Eppley and Myatt, 1998; Gibb, 1998; Brodt-Eppley and Myatt, 1999; Olson, 2003). PGF_2α_ directly stimulates contractions in myometrial smooth muscle via the FP receptor (Karim, 1968; Sharma et al., 1973; Lundström and Bygdeman, 1986; Kelly et al., 2009; Thomas et al., 2014). The binding of PGF_2α_ to the FP receptor activates phospholipase C via Gα_q_, which converts the membrane-bound PIP_2_ to IP_3_ and DAG. The released IP_3_ binds to IP_3_ receptors on the sarcoplasmic reticulum, opening Ca^2+^ channels and increasing cytoplasmic concentrations of Ca^2+^ (Davis et al., 1987; Silvia and Homanics, 1988; Haddock and Hill, 2002). Ca^2+^ binds to calmodulin, which subsequently phosphorylates myosin light-chain kinase, leading to force generation and contraction (Kamm and Stull, 1985; Horowitz et al., 1996; Berridge et al., 2003; Wray and Prendergast, 2019).

In rodents, PGF_2α_ initiates labour by regressing the corpus luteum and reducing systemic progesterone concentrations (Gross et al., 1998). The precise role of PGF_2α_ in active human labour remains undetermined as humans do not experience reductions in systemic progesterone but instead may experience a “functional” progesterone withdrawal (Csapo and Pinto-Dantas, 1965; Merlino et al., 2007). However, maternal plasma levels of PGF_2α_ increase in the third trimester prior to the onset of labour and increase further as labour progresses (reviewed by Wood et al., 2021). Therefore, while PGF_2α_ can stimulate myometrial contractility, it may also play a role in uterine activation and the initiation of parturition.

**FIGURE 1:**
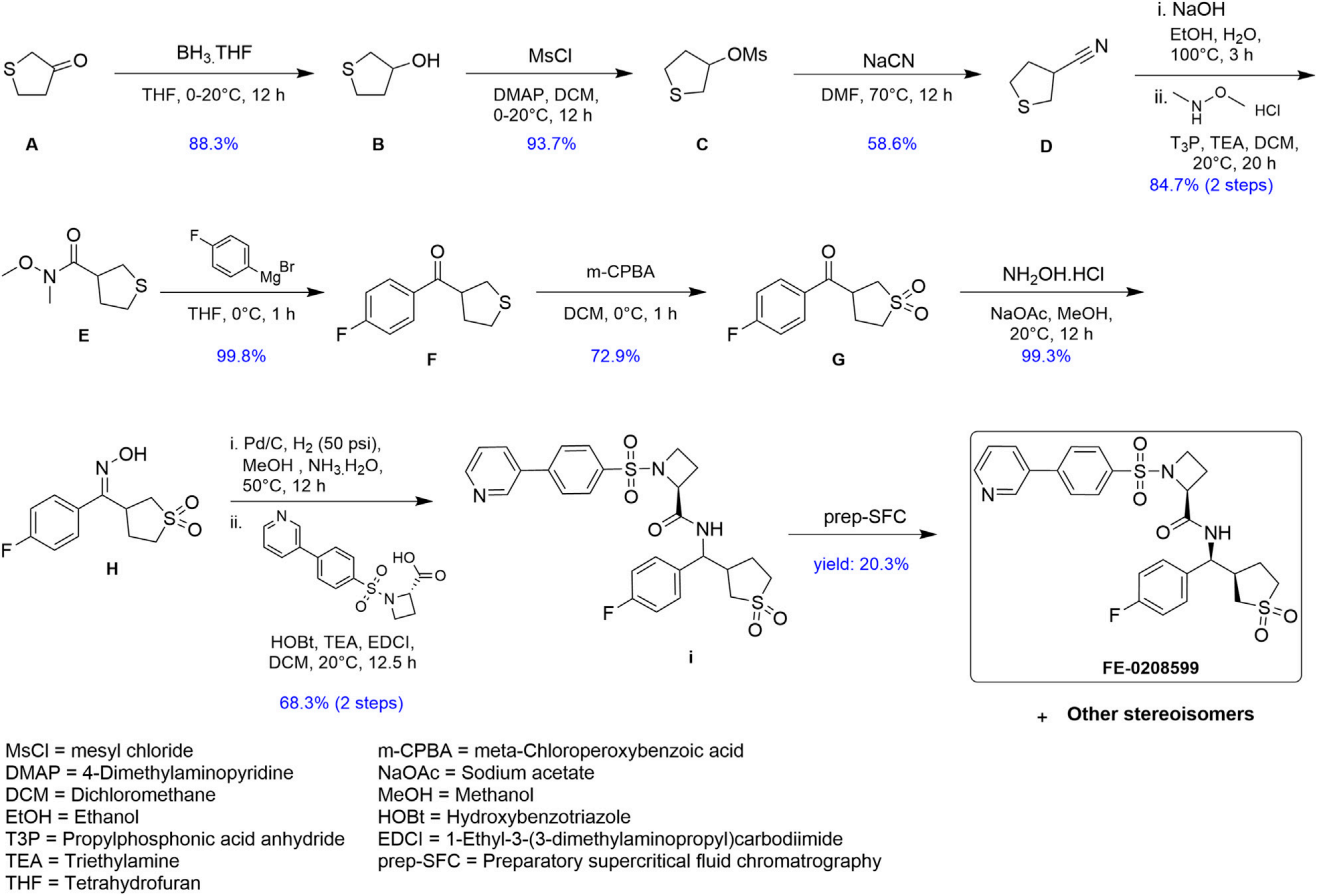
Schematic summary of the synthesis of N582707.

In this study, we used our newly derived myometrial cell line, MYLA, to assess the novel FP antagonist, N582707 (Figure 1), in comparison to four compounds from the literature (Table 1). OBE002 is the parent compound of prodrug OBE022 (ebopiprant), which has been used for the treatment of preterm labour and is currently involved in Phase II clinical trials (Pohl et al., 2019). Compound 46/47 and compound 39/40 were investigated as potential treatments for idiopathic pulmonary fibrosis, with BAY-6672 (derived from compound 46) exhibiting anti-inflammatory and antifibrotic effects in induced pulmonary fibrosis mouse models (Beck et al., 2020). Similarly, compound 68 was designed for the treatment of inflammation and proved to be potent in FLIPR functional assays in transfected HEK-293 cell lines (Martos et al., 2016). We demonstrate that both Ca^2+^ release and Gα_q_ coupling are inhibited by FP antagonists, with the novel FP antagonist, N582707, being more potent when compared to the previously published FP antagonists. Furthermore, through RNA-sequencing (RNA-Seq), we determined that PGF_2α_ causes significant time-dependent changes in mRNA transcription, stimulating a phenotypic switch from a fibroblastic-like phenotype to a smooth muscle-like phenotype with an increase in the number of expressed pro-labour mRNAs. This effect was reversed by FP antagonism. This supports the hypothesis that PGF_2α_ is not only important during labour to stimulate uterine contractions but also plays a significant role in transforming the myometrium from a quiescent to an activated state.

**TABLE 1:** List of FP antagonist chemical names and medical applications.

FP antagonist	Chemical name	Medical application	References
Compound 46/47	5-(6-bromo-3-methyl-2-(pyrrolidin-1-yl) quinoline-4-carboxamido)-4-(2-chlorophenyl)-pentanoic acid	Treatment of idiopathic pulmonary fibrosis	Beck et al. (2020)
Compound 68	3-amino-1-((5-chloro-1-isobutyl-1H-indazol-7-yl) methyl)-1H-indazole-5-carboxylic acid	Treatment of inflammation	Martos et al. (2016)
OBE002	[(S)-3-(biphenyl-4-sulfonyl)-thiazolidine-2-carboxylic acid [(S)-1-(4-fluorophenyl)-3-hydroxy-propyl]-amide]	Treatment of preterm labour	Pohl et al. (2018)
N582707	(S)-N-((S)-((R)-1,1-dioxidotetrahydrothiophen-3-yl)(4-fluorophenyl)methyl)-1-((4-(pyridin-3-yl)phenyl)sulfonyl)azetidine-2-carboxamide	N/A	N/A
Compound 39/40	5-(6-bromo-3-methyl-2-phenylquinoline-4-carboxamido)-4-(2-chlorophenyl) pentanoic acid	Treatment of idiopathic pulmonary fibrosis	Beck et al. (2020)

## Results

**FIGURE 2:**
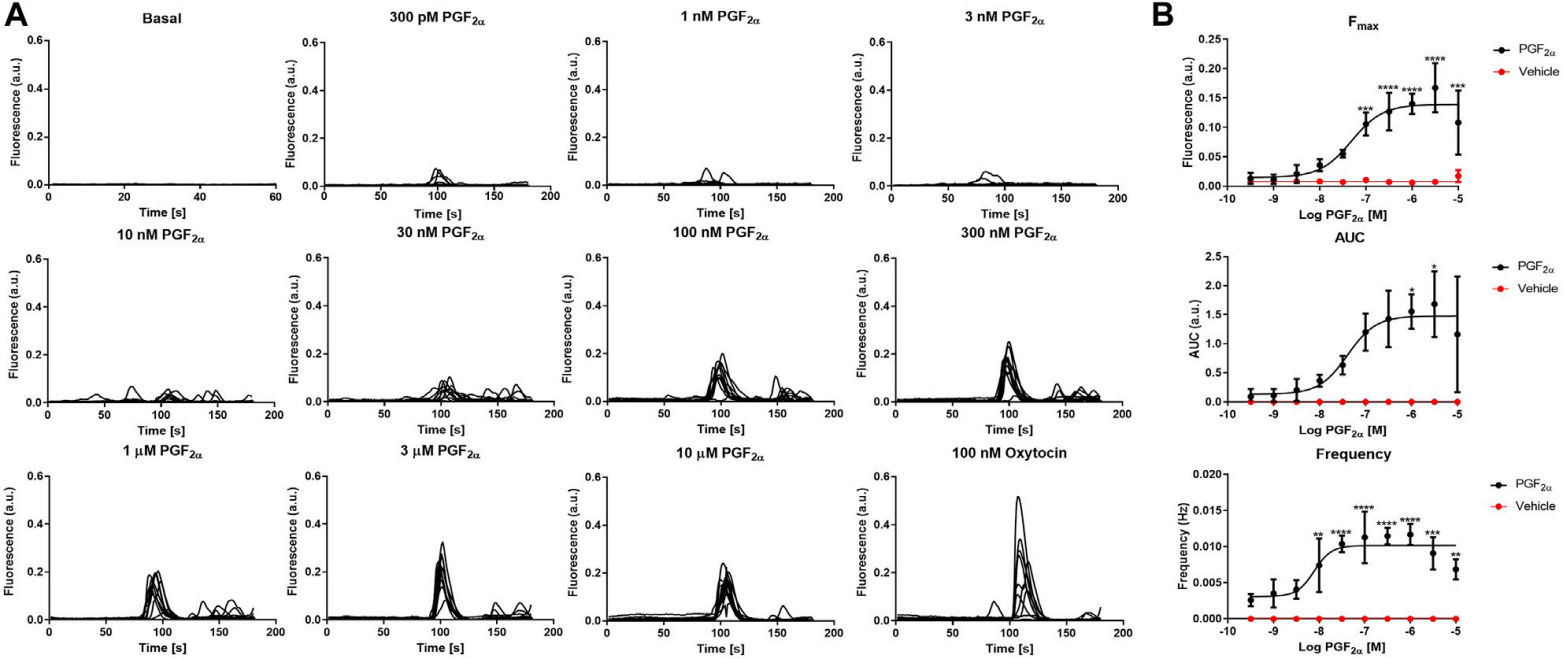
Effect of the FP agonist, PGF_2α,_ upon calcium transients in MYLA cells. **(A)** Representative traces showing calcium transients in MYLA cells in response to increasing concentrations of PGF_2α_ (300 p.m.–10 µM) plus a positive control of 100 nM oxytocin. **(B)** PGF_2α_ (black circles) concentration–response curves and unstimulated/basal (red circles). Data were analysed to assess the peak responses (F_max_), the area under the curve (AUC), and peak frequency (Hz) during the 3 min of PGF_2α_ stimulation. Data calculated as [(raw data − background) – minimum background]/averaged ionomycin F_max_. Data are mean ± SD, N = 3 (data were analysed by one-way ANOVA and Dunnett’s multiple comparison test comparing PGF_2α_ treatment to basal, *p < 0.05, **p < 0.01, ***p < 0.001, and ****p < 0.0001). EC_50_, pEC_50,_ CI, E_max_, and Hill slope values obtained are shown in Table 2. Representative traces for the effect of the PGF_2α_ vehicle (DMSO) are shown in Supplementary Materials S1. Statistical values are shown in Supplementary Materials S2.

To test the basic physiological response of our novel immortalised myometrial cell line (MYLA), we characterised FP receptor signalling by measuring intracellular Ca^2+^ concentrations post PGF_2α_ stimulation. Increasing concentrations of PGF_2α_ (10 half-log incremental concentration; 300 pM to 10 µM) evoked a concentration-dependent increase in the peak height (F_max_) and area under the curve (AUC) of individual intracellular Ca^2+^ oscillations and increased the frequency of Ca^2+^ oscillations as compared to basal values (Figure 2, Supplementary Materials S1, and Supplementary Materials S2). Concentration–response analysis revealed pEC_50_ values of F_max_ 7.442 ± 0.18 (36.11 nM), AUC 7.498 ± 0.21 (31.74 nM) and frequency of 8.572 ± 0.20 (2.678 nM). Confidence intervals (CI), E_max_, and HillSlope values (Table 2) were comparable to data observed in myometrial tissue in response to PGF_2α_ (Phillippe et al., 1997).

**TABLE 2:** Summary statistics for the effect of the FP agonist, PGF_2α,_ upon MYLA cells, as depicted in Figure 1.

300 pM –10 µM PGF_2α_			
Parameters	F_max_	AUC	Frequency
EC_50_	36.11 nM	31.74 nM	2.678 nM
pEC_50_ ± SEM	7.44 ± 0.18	7.59 ± 0.21	8.57 ± 0.20
CI (pEC_50_)	7.80 to 7.08	7.92 to 7.07	8.97 to 8.17
E_max_ ± SEM	0.14 ± 0.01	1.50 ± 0.14	0.01 ± 0.01
HillSlope ± SEM	0.91 ± 0.28	1.06 ± 0.45	0.88 ± 0.33
CI (HillSlope)	0.34 to 1.48	0.13 to 1.99	0.22 to 1.55

Equation = log (agonist) vs. response–variable slope (four parameters) + constrain bottom to 0. *Outliers were identified using Grubb’s (alpha = 0.05).* Data are mean ± SD, N = 3.

### PGF_2α_ stimulates FP receptor coupling to Gα_q/11_ in MYLA cells

**FIGURE 3:**
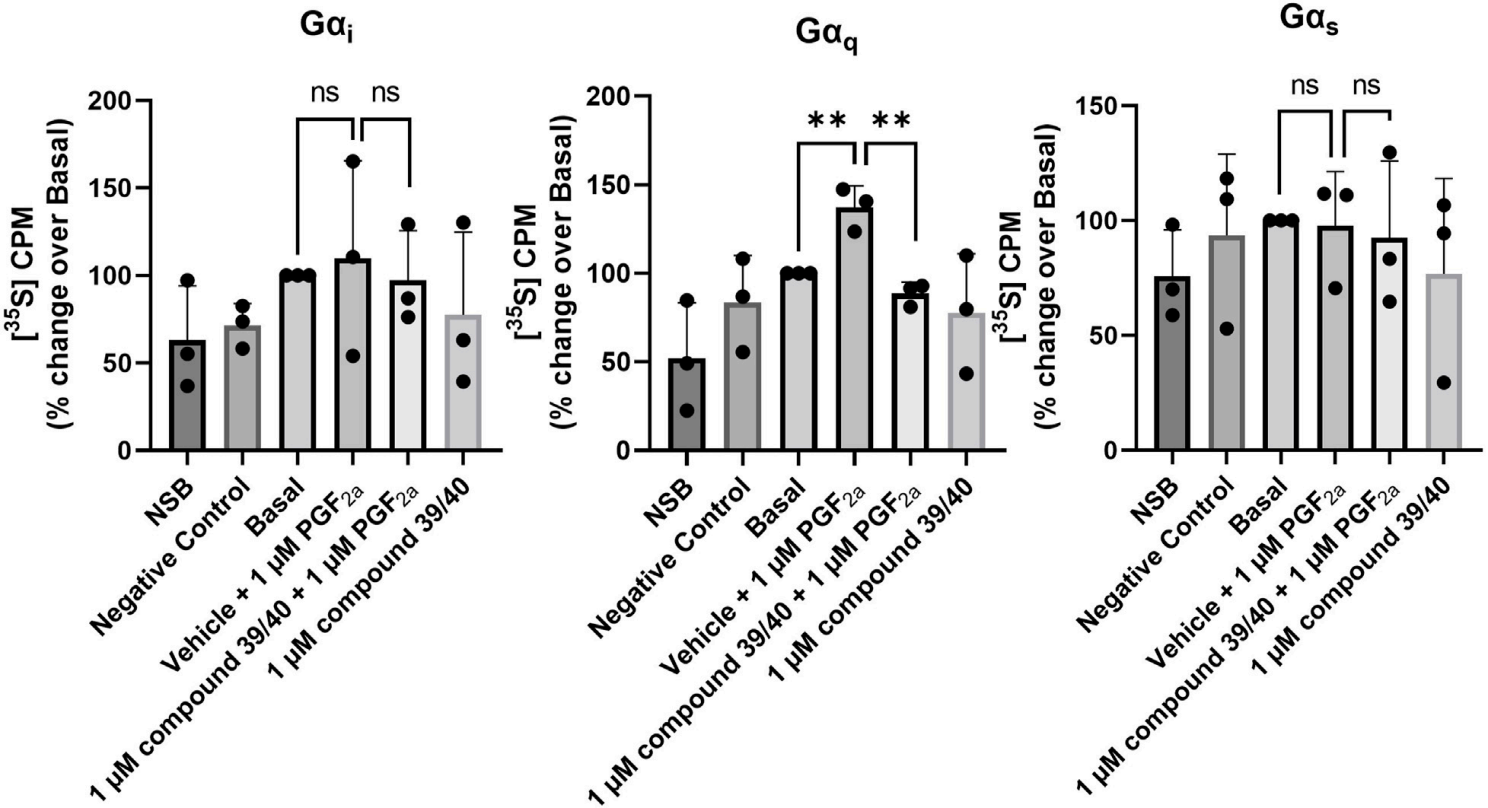
Immunoprecipitation of [^35^S]-GTPγS–bound Gα-protein subunits in MYLA cells. Membranes prepared from cultured MYLA cells, stimulated with PGF_2α_, and inhibited with compound 39/40 for 2 min in the presence of [^35^S]-GTPγS and GDP. Activated membranes were incubated with antisera targeting Gα_q_, Gα_i_, or Gα_s_ G proteins with protein-G Sepharose beads. Non-specific binding (NSB) was determined by incubating with 10 µM unlabelled GTPγS, and a negative control was determined by incubating with isotype control antisera. Data are mean ± SD, N = 3 (data were analysed by one-tailed, unpaired t-tests comparing vehicle + 1 µM PGF_2α_ to basal and vehicle + 1 µM PGF_2α_ to 1 µM compound 39/40 + 1 µM PGF_2α_; ns, not significant; **p < 0.01).

It has previously been demonstrated that the FP receptor couples specifically to the Gα_q_ subunit in mice, rats, and Chinese hamster ovary cells (Davis et al., 1987; Ito et al., 1994; Engstrøm et al., 2000; Le Gouill et al., 2010). To further characterise the MYLA cells, we investigated the coupling of the FP receptor to Gα-subunits by utilising the [35S]-GTPγS immunoprecipitation assay. PGF_2α_ significantly increased [35S]-GTPγS binding to Gα_q/11_ only (Figure 3). However, PGF_2α_ is promiscuous, binding not only to the FP receptor but also to other prostanoid receptors with relatively high affinity (Abramovitz et al., 2000). To demonstrate FP specificity, coupling to Gα_q/11_ was reversed by administration of the FP antagonist compound 39/40.

### The novel FP antagonist, N582707, is more potent than comparator FP antagonists

**FIGURE 4:**
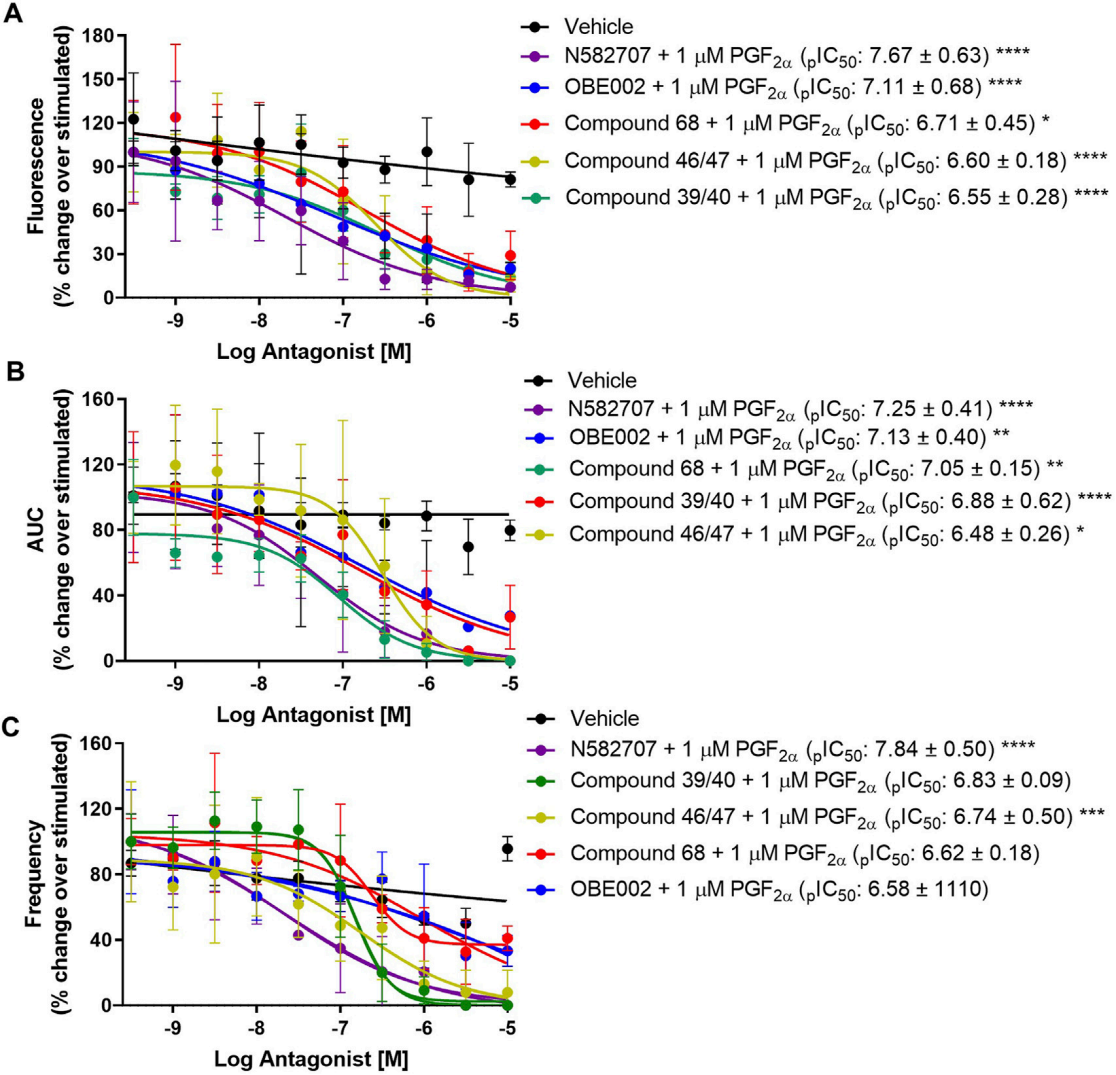
Effect of five FP antagonists upon PGF_2α_-stimulated Ca^2+^ transients in MYLA cells. FP antagonist + 1 µM PGF_2α_ concentration–response curves. Data were analysed to assess **(A)** maximal fluorescence response, **(B)** the area under the curve (AUC), and **(C)** the frequency of Ca^2+^ oscillation during the 3 min of stimulation. Data was calculated as [(raw data − background) − minimum background]/averaged ionomycin F_max_ and then percentage corrected to stimulated (1 µM PGF_2α_). Data are mean ± SD, N = 3. All IC_50_, pIC_50_, CI, and HillSlope values obtained are shown in Table 3. Data were analysed by two-way ANOVA and *post hoc* Dunnett’s multiple comparison test comparing FP antagonist treatment to vehicle, *p < 0.05, **p < 0.01, ***p < 0.001, and ****p < 0.0001.

Using our MYLA cell line, we sought to assess the novel FP antagonist, N582707 (Figure 1), in comparison to four compounds from the literature (Table 1) on PGF_2α_-induced Ca^2+^ oscillations. MYLA cells were incubated with 1 µM PGF_2α_ (to achieve ∼99% FP receptor occupancy and to reflect physiological concentrations) in the presence of 10 half-log incremental concentrations: 300 pM to 10 µM of FP antagonists. The results demonstrated that the FP antagonists inhibited PGF_2α_-stimulated Ca^2+^ release as determined by F_max_, AUC, and frequency (Figure 4; Table 3). Of the five tested FP antagonists, N582707 was the most potent for all tested criteria, with pIC_50_ values in a nanomolar range [F_max_ 7.67 ± 0.63 (IC_50_ 21.26 nM), AUC 7.30 ± 0.32 (IC_50_ 50.43 nM) and frequency of Ca^2+^ oscillations 7.66 ± 0.41 (IC_50_ 22.15 nM)].

**TABLE 3:** Summary statistics for the effect of the five FP antagonists upon MYLA cells, as depicted in Figure 4.

All antagonists in the presence of 1 µM PGF2α					
Effect on F_max_					
Parameters	300 pM–10 µM compound 46/47	300 pM–10 µM compound 68	300 pM–10 µM OBE002	300 pM–10 µM N582707	300 pM–10 µM compound 39/40
IC_50_	249.80 nM	193.60 nM	78.32 nM	21.26 nM	283.80 nM
pIC_50_ ± SD	6.60 ± 0.18	6.71 ± 0.45	7.11 ± 0.68	7.67 ± 0.63	6.55 ± 0.28
CI (pIC_50_)	6.98 to 6.2	7.64 to 5.79	8.507 to 5.71	8.96 to 6.38	7.12 ± 5.97
HillSlope ± SD	−1.02 ± 0.40	−0.47 ± 0.19	−0.38 ± 0.15	−0.49 ± 0.20	−0.54 ± 1.63
CI (HillSlope)	−1.84 to −0.21	−0.86 to 0.09	−0.68 to 0.07	−0.89 to 0.08	−0.88 ± −0.21
Effect on AUC					
Parameters	300 pM–10 µM compound 46/47	300 pM–10 µM compound 68	300 pM–10 µM OBE002	300 pM–10 µM N582707	300 pM–10 µM compound 39/40
IC_50_	299.4 nM	174.9 nM	179.7 nM	50.43 nM	89.95 nM
pIC_50_ ± SD	6.52 ± 0.18	6.76 ± 0.52	6.75 ± 0.57	7.30 ± 0.32	7.05 ± 0.15
CI (pIC_50_)	6.89 to 6.16	7.83 to 5.68	7.93 to 5.56	7.96 to 6.64	7.35 ± 6.74
HillSlope ± SD	−1.37 ± 0.68	−0.44 ± 0.19	−0.41 ± 0.17	0.69 ± 0.27	−0.98 ± 0.27
CI (HillSlope)	−2.78 to −0.04	−0.84 to −0.05	−0.76 to −0.05	−1.24 to −0.14	−1.54 ± 0.41
Effect on frequency					
Parameters	300 pM–10 µM compound 46/47	300 pM–10 µM compound 68	300 pM–10 µM OBE002	300 pM–10 µM N582707	300 pM–10 µM compound 39/40
IC_50_	175.2 nM	977.7 nM	1975 nM	22.15 nM	154.0 nM
pIC_50_ ± SD	6.76 ± 0.32	6.01 ± 0.30	5.70 ± 0.48	7.66 ± 0.41	6.81 ± 0.08
CI (pIC_50_)	7.42 to 6.09	6.62 to 5.40	6.69 to 4.72	8.50 to 6.81	6.97 ± 6.66
HillSlope ± SD	−0.69 ± 0.30	−0.49 ± 0.17	−0.36 ± 0.19	−0.55 ± 0.17	−2.00 ± 0.56
CI (HillSlope)	−1.31 to −0.08	−0.84 to −0.13	−0.74 to 0.03	−0.90 to −0.19	−3.15 ± −0.86

Equation = log (inhibitor) vs. response–variable slope (four parameters) + constrain bottom to 0. Outliers were identified using Grubb’s (alpha = 0.05). Data are mean ± SD, N = 3.

### PGF_2α_ stimulates the development of a pro-labour phenotype and induces a phenotypical switch from a myofibroblastic to a smooth muscle phenotype in MYLA cells

**FIGURE 5:**
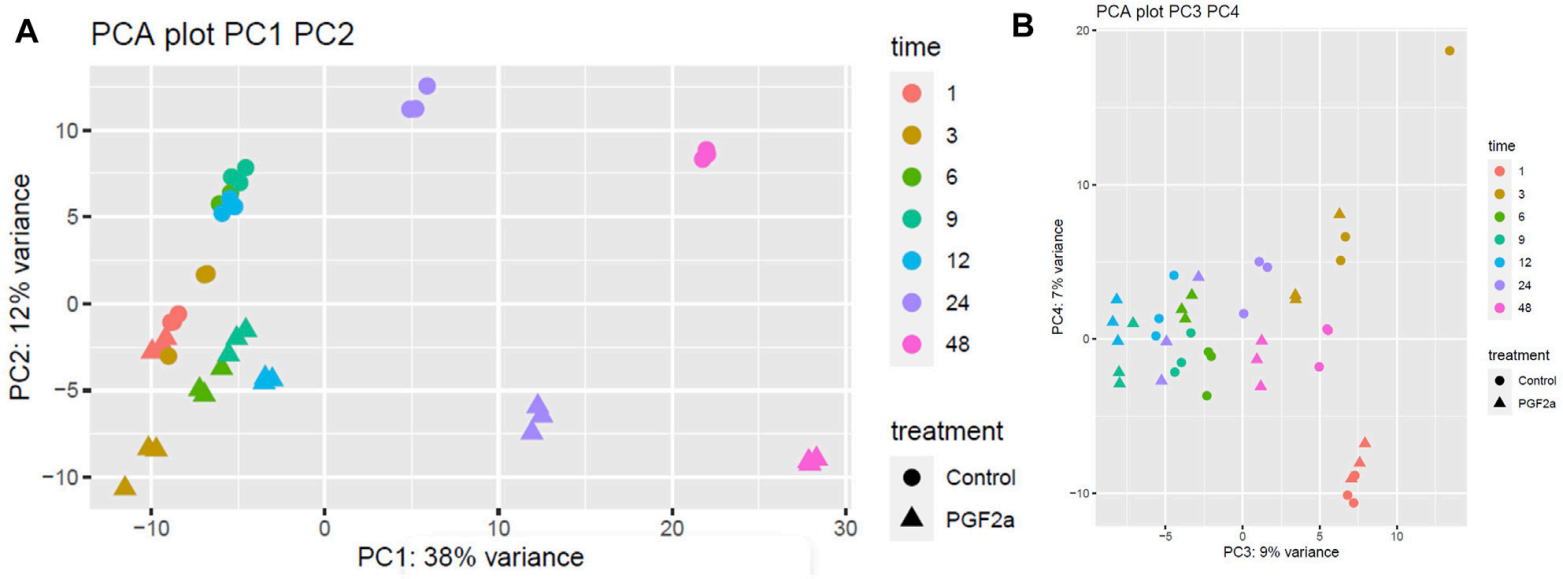
PCA plots of RNA-Seq data to capture the largest sources of variability in MYLA transcriptomes. RNA-Seq data was collected from MYLA cells treated with either 1 µM PGF_2α_ or vehicle equivalent at time 0 followed by cells harvested at 1 h, 3 h, 6 h, 9 h, 12 h, 24 h, and 48 h. Colours indicate different time points, and shapes indicate different treatments; control (circles) or PGF_2α_ (triangles). **(A)** Principal component analysis (PCA) plot PC1 captures a difference in grouping by time defined as ‘early’ (1 h, 3 h, 6 h, 9 h, and 12 h) or ‘late’ (24 h and 48 h). PC2 captures a difference in grouping by treatment (control vs. PGF_2α_). In total, PC1 and PC2 capture 50% of transcriptomic variation. **(B)** PCA plot PC3 captures a second grouping by time, whereby ‘early’ (1 h and 3 h) and ‘late’ (24 h and 48 h) time points are grouped, and ‘mid’ time points (6 h, 9 h, and 12 h) are grouped.

**FIGURE 6:**
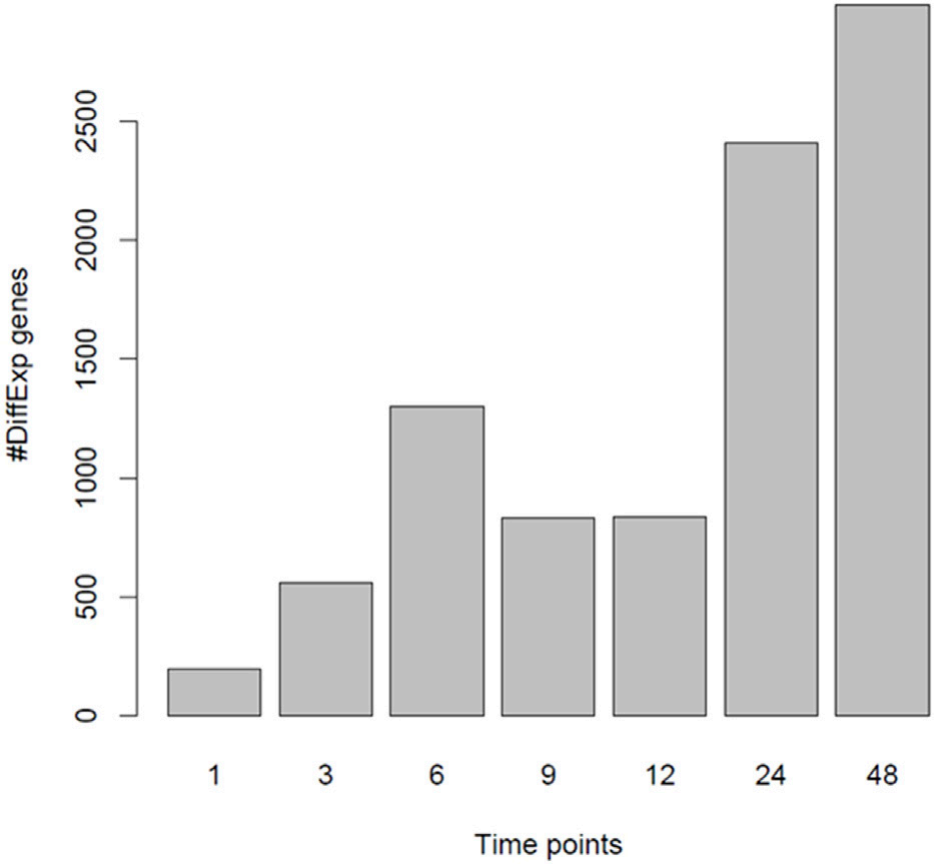
Number of differentially expressed genes when comparing PGF_2α_ vs. control data. RNA-Seq data was collected from MYLA cells treated with either 1 µM PGF_2α_ or vehicle equivalent at time 0 followed by harvesting cells at 1 h, 3 h, 6 h, 9 h, 12 h, 24 h, and 48 h. The number of differentially expressed genes increased with time in two phases, 1 h to 6  h and then from 24 h to 48 h.

It has previously been reported that PGF_2α_ is not only involved in the contractile phase of labour but also in the activation of parturition (Xu et al., 2013; Xu et al., 2015). To assess if PGF_2α_ stimulates the development of a pro-labour phenotype, MYLA cells were treated with either 1 µM PGF_2α_ or ethanol vehicle control and then harvested at seven time points after treatment. Principal component analysis (PCA) plot PC1 (Figure 5A) demonstrates a difference in grouping by time, defined as ‘early’ (1 h, 3 h, 6 h, 9 h, and 12 h) or ‘late’ (12 h and 24 h). PC2 captures a difference in grouping by treatment (control vs. PGF_2α_). Together, PC1 and PC2 demonstrate that the data were clean and clustered well, describing 50% of transcriptomic variation. PCA plot PC3 (Figure 5B) captured a second grouping of mRNA changes by time, whereby ‘early’ (1 h and 3 h) and ‘late’ (24 h and 48 h) time points are grouped, and ‘mid’ (6 h, 9 h, and 12 h) time points are grouped. Differential gene expression analysis showed that with an increase in time, there was an increase in the number of differentially expressed genes (Figure 6).

**FIGURE 7:**
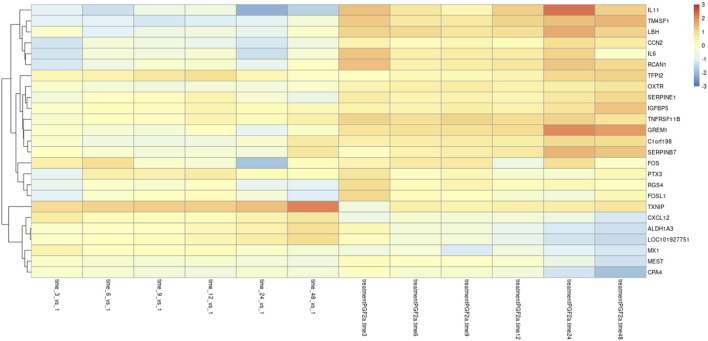
Differential gene expression in MYLA transcriptomes. RNA-Seq data was collected from MYLA cells treated with either 1 µM PGF_2α_ or vehicle equivalent at time 0 followed by harvesting cells at 1 h, 3 h, 6 h, 9 h, 12 h, 24 h, and 48 h. Heatmap of the top 25 differentially expressed genes in the control and PGF_2α_-treated cells as compared to their 1 h time point, identified using DESeq2.

To investigate further changes in the mRNA levels, we generated Z-scores of the transcripts per million (TPM) values for the >2,500 differentially expressed genes. A heat map of the top 25 most significant differentially expressed genes as compared to their 1-h time point is depicted in Figure 7. As suggested by the PCA, there was a clear separation between PGF_2α_ treatment and vehicle control. Several key genes involved in labour, such as *OXTR*, were upregulated in the PGF_2α_ treatment group as well as upregulated over time. Also prominent was an upregulation of pro-labour genes that are associated with leucocyte infiltration and inflammation such as *IL6* and *IL11*. Conversely, several genes such as *CXCL12*, *ALDH1A3*, and *CPA4* decreased in expression with time and treatment.

The RNA-Seq data were then cross-referenced with a publicly available data set (WikiPathways 2021 human) to determine the top 10 enrichment terms (Table 4). The most enriched term was ‘myometrial relaxation and contraction,’ suggesting that PGF_2α_ alone was stimulating the development of genes associated with a pro-labour phenotype as well as a smooth muscle phenotype (e.g., *CALD1* and *ACTA2*). The RNA-Seq data were then cross-referenced with the data set generated by Chan et al. (2014) that measured the transcriptome differences in human myometrial samples prior to and after the onset of spontaneous labour (Table 5). Comparatively, over time, the number of differentially expressed genes per time point in both data sets increased from 26 to 141 common genes and smooth muscle markers from 2 to 18 common genes, implicating again that PGF_2α_ alone was stimulating not only a pro-labour phenotype but also the MYLA cells to differentiate into a smooth muscle phenotype.

**TABLE 4:** Top 10 most significant enrichment terms and genes expressed within those terms as defined by WikiPathways 2021 human.

Enrichment term	*p*-value	Genes within the enrichment term
Myometrial relaxation and contraction pathways WP289	3.954e-07	*OXTR*, *IGFBP5*, *IGFBP4*, *ATP2A2*, *ADM*, *ACTB*, *ADCY6*, *ACTA2*, *RGS5*, *CNN1*, *RGS2*, *ACTC1*, *CALD1*, *IL1B*, *RAMP1*, *ATF3*, and *RGS7*
Cholesterol biosynthesis pathway WP197	9.789e-07	*FDPS*, *CYP51A1*, *MSMO1*, *HMGCR*, *DHCR7*, and *FDFT1*
Senescence and autophagy pathway WP615	1.239e-05	*MAP2K3*, *VTN*, *UVRAG*, *GABARAPL1*, *MMP14*, *IGFBP5*, *IL1B*, *SERPINE1*, *IL24*, *E2F1*, *INHBA*, and *THBS1*
VEGFA-EGRF2 signalling pathways WP3888	1.352e-05	*KANK1*, *HDAC5*, *NRP2*, *FLT1*, *CYR61*, *CTGF*, *ICAM1*, *CSRP2*, *PNP*, *PTPRZ1*, *CCND1*, *ADAMTS1*, *CCL2*, *CHAC1*, *MAP2K3*, *DUSP5*, *HSP90AA1*, *PLAUR*, *SHROOM2*, *PDIA6*, *RCAN1*, *NR4A1*, *MMP14*, *NR4A3*, *HYOU1*, *P4HB*, and *PTMA*
Cholesterol metabolism (includes both Bloch and Kandutsch–Russell pathways) WP4718	1.633e-05	*FDPS*, *CH25H*, *FASN*, *CYP51A1*, *MSMO1*, *HMGCR*, *DHCR7*, and *FDFT1*
Lung fibrosis WP3624	2.532e-05	*GREM1*, *FGF7*, *MT2A*, *CCL11*, *ELN*, *IL1B*, *CCL2*, *FGF1*, and *CTGF*
Copper homeostasis WP3286	4.129e-05	*MT2A*, *CCND1*, *SCO1*, *SLC31A1*, *MT1X*, *STEAP1*, *STEAP2*, and *MT1E*
Focal adhesion WP306	1.438e-04	*FLT1*, *ACTN4*, *THBS1*, *ACTB*, *MYL12A*, *MYLK*, *MYL12B*, *VTN*, *RELN*, *CCND1*, *COL4A1*, *PDGFD*, *COL5A2*, *ITGA8*, and *ITGA7*
IL-18 signalling pathway WP4754	1.814e-04	*PHF20*, *TNFRSF11B*, *ICAM1*, *NPPB*, *ACTA2*, *CCNA2*, *SLC4A7*, *NR4A1*, *MMP14*, *PTPRZ1*, *IL1B*, *CCL2*, *ULBP2*, *ATF3*, *SNTB1*, *IER3*, *AARS*, and *TGM2*
Focal adhesion PI3K-Akt-mTOR signalling pathway WP3932	2.419e-04	*HSP90AA1*, *FLT1*, *COL11A1*, *IRS2*, *SLC2A3*, *FGF1*, *HIF1A*, *THBS1*, *HSP90B1*, *VTN*, *TBC1D1*, *FGF7*, *RELN*, *COL4A1*, *PDGFD*, *COL5A2*, *ITGA8*, *ITGA7*, and *IL7R*

**TABLE 5:** Number of common differentially expressed genes per time point in our RNA-Seq data and the Chan et al. (2014) data set.

Time point (hours)	1	3	6	12	24	48
Number of common genes upregulated with labour	26	65	96	71	139	141
Number of common genes downregulated with labour	8	19	51	43	84	10
Number of smooth muscle markers	2	1	12	12	18	14

### FP antagonism prevents PGF_2α_-stimulated activation of the myometrium

**FIGURE 8:**
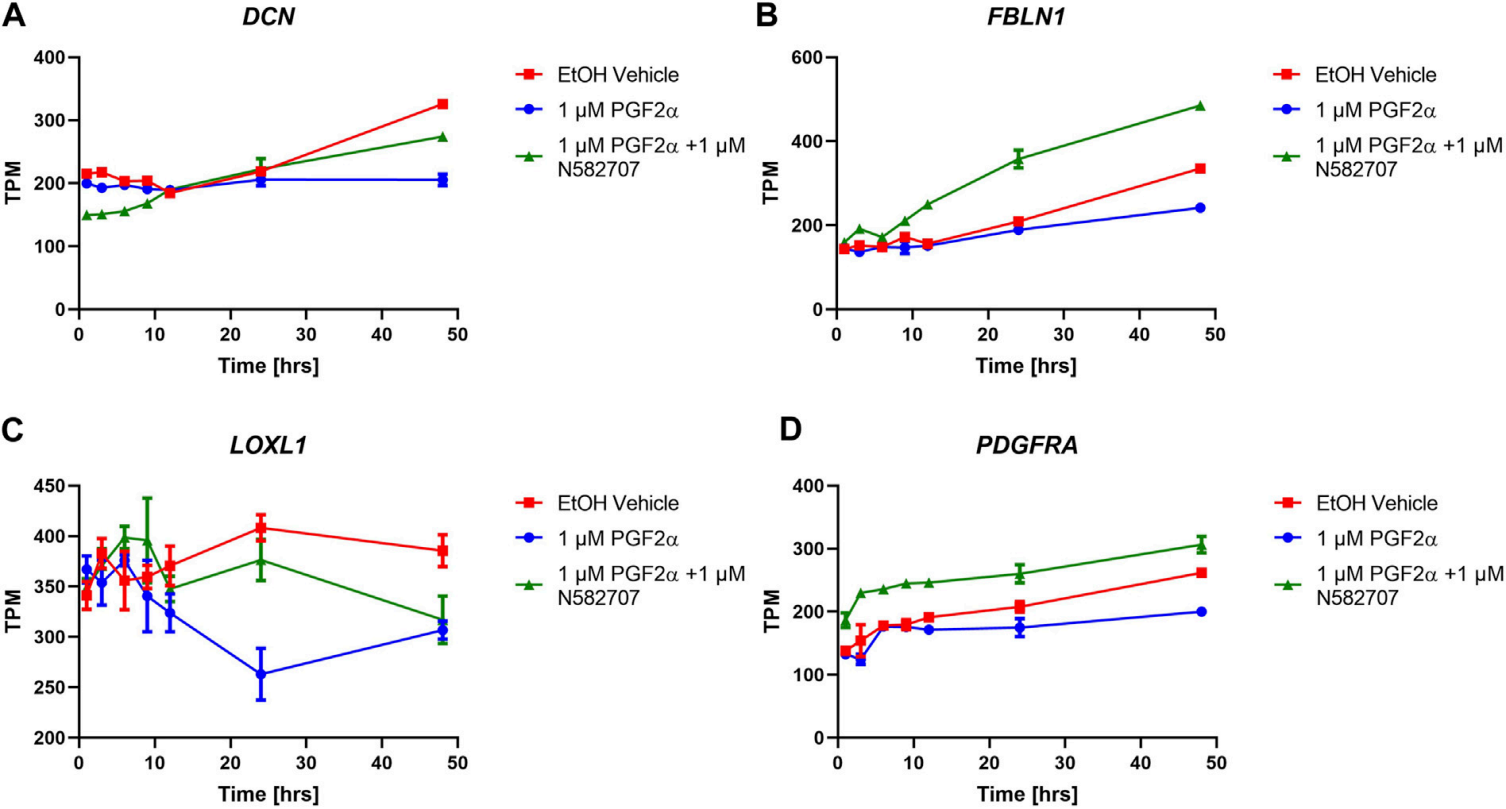
Temporal changes in selected fibroblast markers over a 48-h period in FP agonist and FP antagonist–treated MYLA cells. MYLA cells were stimulated for 1, 3, 6, 9, 12, 24, and 48 h with 1 µM PGF_2α_ (blue), EtOH vehicle (red), or 1 µM PGF_2α_ and 1 µM N582707 (green). **(A)** *DCN* **(B)** *FBLN1*, **(C)** *LOXL1*, and **(D)** *PDGFRA*. Expressions of the four fibroblast marker genes were analysed over time as determined by RNA-Seq and expressed as transcripts per million (TPM). Each time point was performed in triplicate and is represented as mean ± SD. Data were analysed by two-way ANOVA and Dunnett’s multiple comparison test comparing treatments to EtOH vehicle, *p < 0.05, **p < 0.01, and ****p < 0.0001.

**FIGURE 9:**
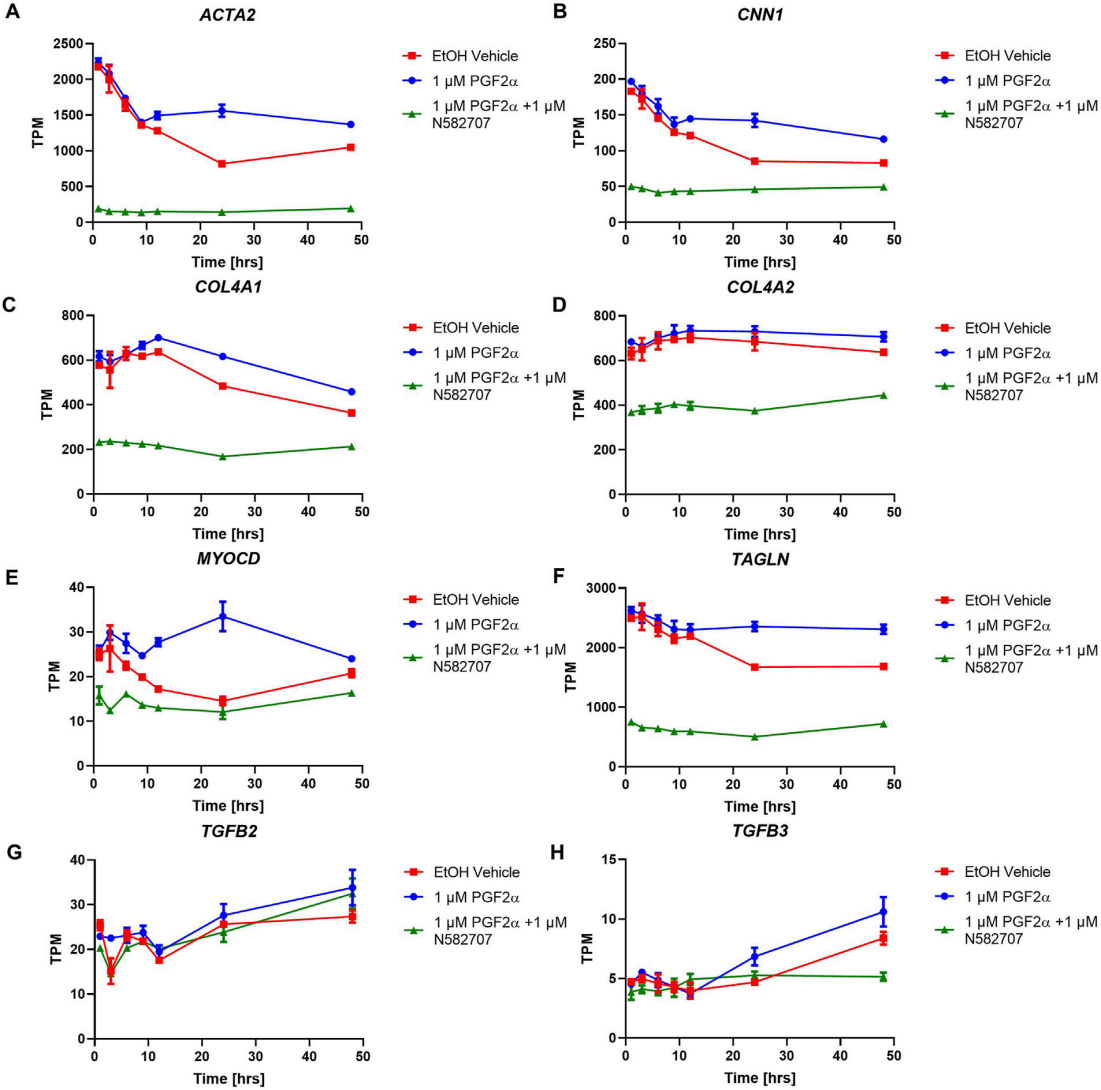
Temporal changes in selected smooth muscle markers over a 48-h period in FP agonist and FP antagonist–treated MYLA cells. MYLA cells were stimulated for 1, 3, 6, 9, 12, 24, and 48 h with 1 µM PGF_2α_ (blue), EtOH vehicle (red), or 1 µM PGF_2α_ and 1 µM N582707 (green). **(A)** *ACTA2*, **(B)** *CNN1*, **(C)** *COL4A1*, **(D)** *COL4A2*, **(E)** *MYOCD*, **(F)** *TAGLN*, **(G)** *TGFB2*, and **(H)** *TGFB3*. Expressions of the eight smooth muscle marker genes were analysed over time as determined by RNA-Seq and expressed as transcripts per million (TPM). Each time point was performed in triplicate and is represented mean ± SD. Data were analysed by two-way ANOVA and Dunnett’s multiple comparison test comparing treatments to EtOH vehicle, *p < 0.05, **p < 0.01, and ****p < 0.0001.

**FIGURE 10:**
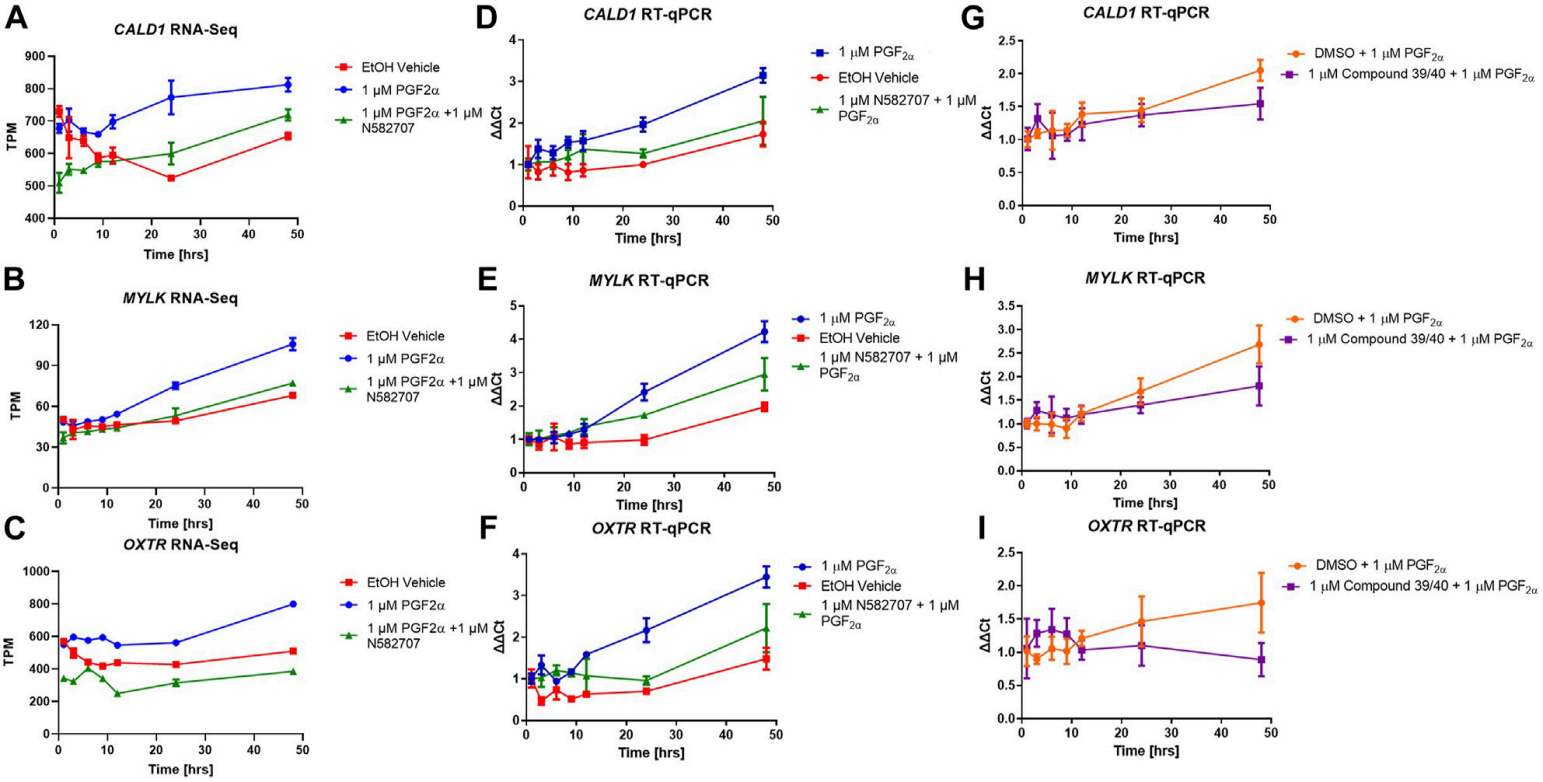
Transcriptional changes in FP agonist and FP antagonist–treated MYLA cells during a 48-h period as determined by RNA-Seq and RT-qPCR. MYLA cells were stimulated for 1, 3, 6, 9, 12, 24, and 48 h with 1 µM PGF_2α_ (blue), EtOH vehicle (red), or 1 µM PGF_2α_ and 1 µM N582707 (green). **(A)** *CALD1* and **(B)** *MYLK* were used as representative smooth muscle cell markers, and **(C)** *OXTR* as a pro-labour marker. Expressions of the three genes were analysed over time as determined by RNA-Seq and expressed as transcripts per million (TPM). **(D)** *CALD1*, **(E)** *MYLK*, and **(F)** *OXTR* expressions over time as determined by RT-qPCR and expressed as fold change (ΔΔCt) using the geometric mean of *RPL19*, *GAPDH*, and *ACTB* housekeeping genes and as compared to equivalent 1 h time points. In a second time series, MYLA cells were stimulated for 1, 3, 6, 9, 12, 24, and 48 h with 1 µM DMSO + 1 µM PGF_2α_ (orange) or 1 µM PGF_2α_ and 1 µM compound 39/40 (purple). **(G)** *CALD1*, **(H)** *MYLK*, and **(I)** *OXTR* expressions over time as determined by RT-qPCR expressed as fold change (ΔΔCt) using the geometric mean of *RPL19*, *GAPDH*, and *ACTB* housekeeping genes and as compared to equivalent 1 h time points. Each time point was performed in triplicate and is represented as mean ± SD. Data were analysed by two-way ANOVA and Dunnett’s multiple comparison test comparing treatments to EtOH vehicle control, *p < 0.05, **p < 0.01, ***p < 0.001, and ****p < 0.0001.

**FIGURE 11:**
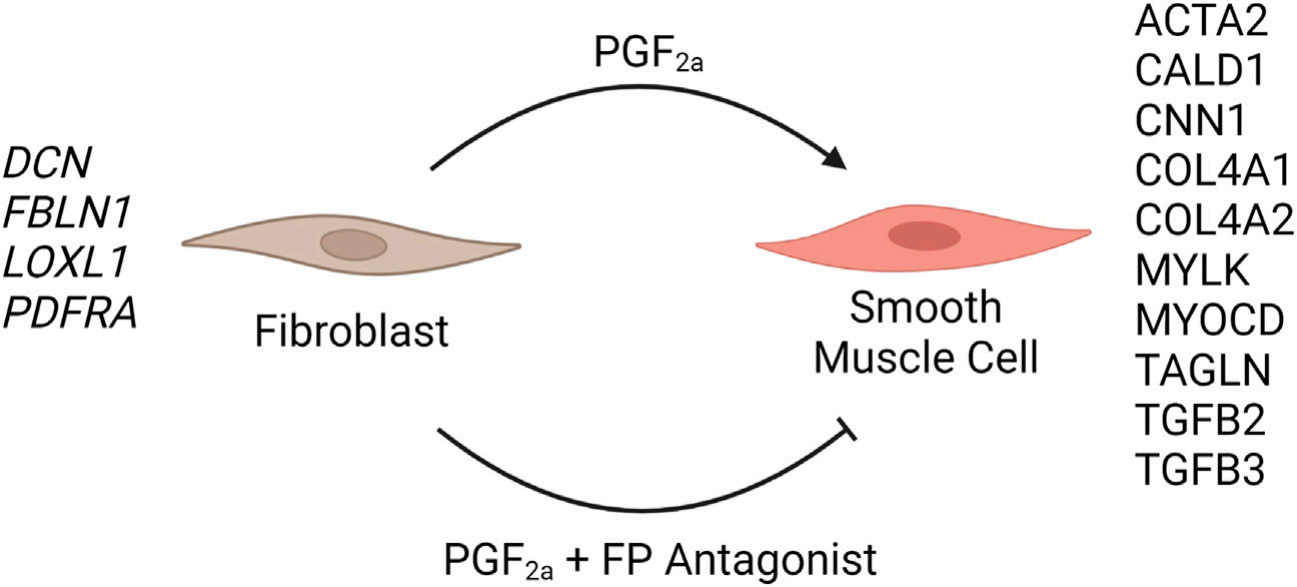
Schematic summary of the effect of PGF_2a_-treated or PGF_2a_ + FP antagonist–treated MYLA cells. The addition of 1 µM PGF_2α_ to MYLA cells stimulated increased expression of several smooth muscle marker genes, namely, *ACTA2*, *CALD1*, *CNN1*, *COL4A1*, *COL4A2*, *MYLK*, *MYOCD*, *TAGLN*, *TGFB2*, and *TGGFB3*. The addition of 1 µM PGF_2α_ + 1 µM N582707 increased the expression of several fibroblast marker genes including *DCN*, *FBLN1*, *LOXL1*, and *PDFRA*.

To determine that the development of a pro-labour phenotype and a smooth muscle phenotype is specifically FP receptor-mediated, additional time series data were conducted using MYLA cells treated with 1 µM PGF_2α_ or 1 µM PGF_2α_ + 1 µM N582707 or 1 µM PGF_2α_ + 1 µM compound 39/40. Four fibroblast marker genes (*DCN*, *LOXL1*, *FBLN1*, and *PDGFRA*—Figure 8) and 10 smooth muscle marker genes (*ACTA2*, *CNN1*, *COL4A1*, *COL4A2*, *MYOCD*, *TAGLN*, *TGFB2*, *TGFB3*—Figure 9; *CALD1* and *MYLK*—Figure 10) were analysed. The expression of all 10 smooth muscle markers was lower in both the control and FP antagonist–treated groups, while the expression of the four fibroblastic markers was higher in the control and FP antagonist–treated groups, as summarised in Figure 11. This indicates that PGF_2α_ initiates a phenotypical switch in the MYLA cells, whereby they undergo differentiation from a myofibroblastic to a smooth muscle phenotype via FP receptor signalling.

**FIGURE 12:**
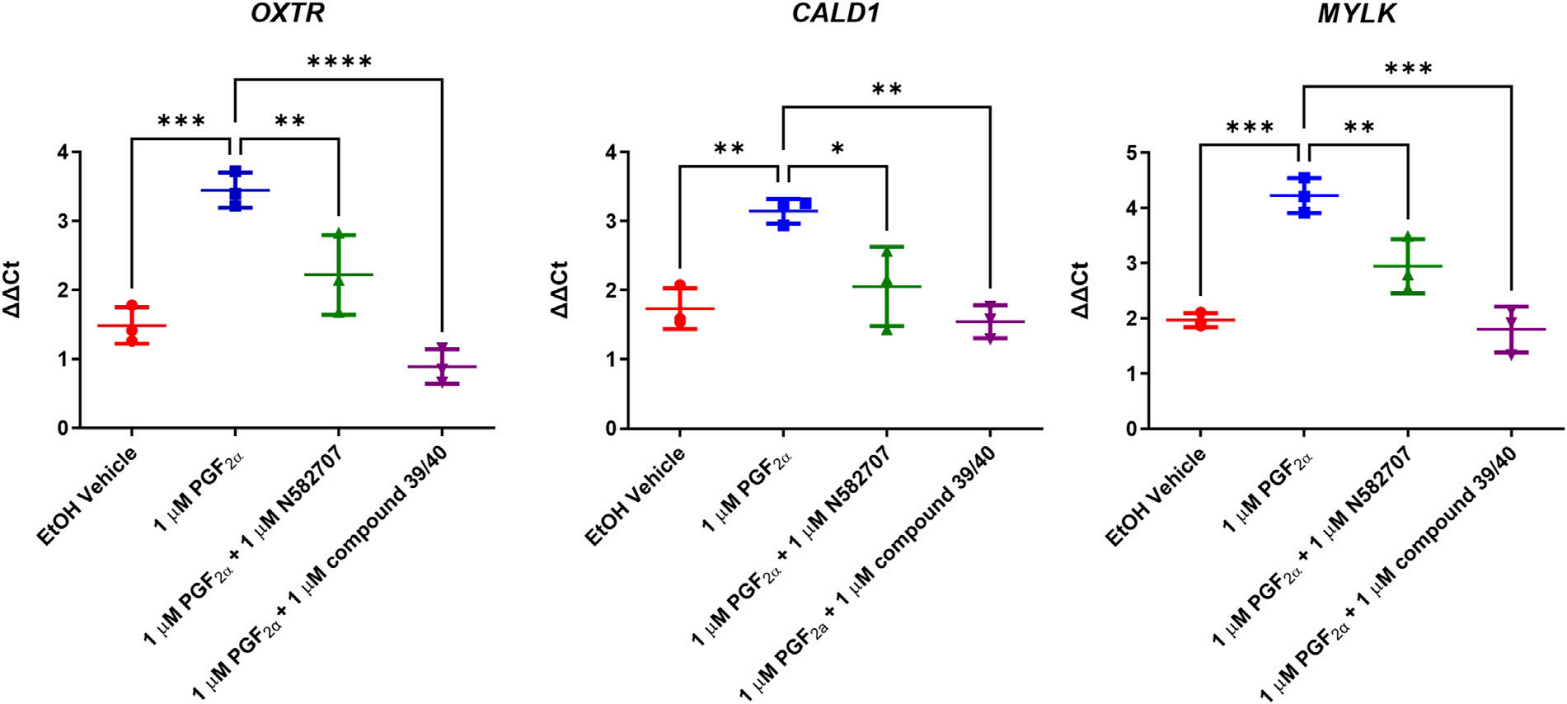
Expressions of *OXTR*, *CALD1*, and *MYLK* in MYLA immortalised myometrial cells after 48 h treatment with PGF_2α_ or PGF_2α_ + FP antagonist. Cells were stimulated for 48 h with vehicle, 1 µM PGF_2α_ and 1 µM PGF_2α_ + 1 µM N582707 or compound 39/40. *CALD1* and *MYLK* were used as representative smooth muscle cell markers and *OXTR* due to their association with the progression of labour. Data are expressed as fold change (ΔΔCt). Data are mean ± SD, N = 3, one-way ANOVA and *post hoc* Dunnett’s multiple comparison test comparing conditions to 1 µM PGF_2α_, *p < 0.05, **p < 0.01, ***p < 0.001, and ****p < 0.0001.

The pro-labour gene, *OXTR*, and two smooth muscle markers, *CALD1* and *MYLK*, were used to validate these results using RT-qPCR. This determined that both N582707 and compound 39/40 reduced the expression of *OXTR*, *CALD1*, and *MYLK* (Figure 10). When specifically looking at the difference between gene expression at 48 h, there was a significant decrease in *OXTR*, *CALD1*, and *MYLK* expression when treated with either N582707 or compound 39/40 (Figure 12). Overall, this demonstrates that PGF_2α_ via the FP receptor could initiate activation of the myometrium prior to labour and implies a potential role for FP antagonism in the prophylactic management of PTB in addition to the known effects on uterine contractility.

## Discussion

It is commonly accepted that PGF_2α_ is involved in several events during human parturition, such as stimulating cervical ripening, rupturing the foetal membranes, and playing an important role during the final stages of parturition by regulating uterine contractility (Maclennan and Green, 1979; Casey and MacDonald, 1988; Senior et al., 1993; Lee et al., 2009). While evidence exists that PGF_2α_ can upregulate the expression of uterine activation proteins, an in-depth analysis of the effect of PGF_2α_ upon the myometrium is yet to be elucidated (Xu et al., 2013; Xu et al., 2015). Furthermore, the FP receptor is expressed in a limited number of human tissues (eye and myometrium) (Matsumoto et al., 1997; Mukhopadhyay et al., 2001) and is involved in pulmonary fibrosis (Oga et al., 2009). This makes the FP receptor an attractive target for the development of novel therapies for PTB.

This study was a first look at several comparator FP antagonists, of which only OBE002 has been investigated for the treatment of PTB, in comparison to a novel FP antagonist, N582707. We demonstrated that our MYLA cell line derived from myometrial tissue obtained from a pregnant, non-labouring woman was a suitable human myometrial model. Stimulation with PGF_2α_ activated Gα_q_-specific G-protein coupling with no effect on non-FP receptor–specific Gα_s_ and Gα_i_ coupling in addition to a concentration-dependent increase in intracellular Ca^2+^. Both Gα_q_ coupling and Ca^2+^ release were inhibited by FP antagonists, with the novel FP antagonist N582707 being the most potent when compared to several known FP antagonists. This highlighted the potential of N582707 to be used as a tocolytic treatment for PTB management.

Prior to the onset of labour, the myometrium must undergo a process of activation, whereby the muscle becomes more electrically excitable and susceptible to pro-contractile hormones (Blanks and Brosens, 2012). This is mediated by an increased expression of contraction-associated genes such as *OXTR*, *PTGS2*, and *CX43* (Blanks and Brosens, 2012; Xu et al., 2013). Therefore, in addition to a tocolytic effect, we sought to determine if PGF_2α_ could initiate activation of the myometrium and, furthermore, if treatment with N582707 could inhibit this activation. Using RNA sequencing, we determined that PGF_2α_ stimulates a time-dependent phenotypic transformation of MYLA cells from a myofibroblast-like phenotype to a smooth muscle-like phenotype and increases the number of pro-labour genes. When cross-referencing our transcriptomics data to fresh human samples from term not in labour and spontaneous labour from data collected by Chan et al. (2014), we found a significant overlap in the mRNA signature seen in our PGF_2α_-stimulated MYLA time course. FP antagonism inhibited this phenotypical switch, supporting the hypothesis that PGF_2α_ not only is important during labour to stimulate contractions but also plays a critical role in genetically transforming the myometrium from a quiescent to an activated state. Therefore, these data demonstrate that in addition to using FP antagonists as tocolytics, FP antagonism could be used prophylactically to prevent the maturation of the myometrial smooth muscle to a pro-labour phenotype.

While this time series provided insight into the changes elicited by PGF_2α_ in MYLA cells, we did not include an analysis on the mRNA transcriptome in the presence of progesterone. In a study by Madsen et al. (2004), it was determined that prostaglandins such as PGF_2α_ have the potential to induce functional progesterone withdrawal by modulating progesterone receptor isoform expression. Therefore, it would be beneficial to develop this labour model by the addition of other hormones such as progesterone to more closely simulate the changes that occur during pregnancy. Furthermore, in future studies, it will be important to demonstrate functional changes in the myometrial cell phenotype that are reversible with FP antagonism.

We hypothesise that in addition to the importance of PGF_2α_ in the progression of labour, PGF_2α_ may also be important in the activation of the myometrium by inducing a phenotypical switch in myometrial cells, causing the development of a smooth muscle phenotype and upregulating pro-labour genes. This suggests not only a therapeutic potential for FP receptor antagonists as a tocolytic treatment for PTB but also the potential of using FP antagonists prophylactically to prevent premature activation of the myometrium.

## Materials and methods

### Cell culture

Primary myometrial cells (MYLA) were established from a myometrial sample obtained with informed consent from a pregnant woman at 38 weeks gestation undergoing elective caesarean section for breech presentation at term not in labour. Following delivery of the baby and prior to delivery of the placenta, a full-thickness myometrial biopsy was taken, prior to Syntocinon bolus, from the upper lip of the lower uterine segment incision in the midline. The sample was placed in modified Krebs–Henseleit solution (mM): NaCl, 133; KCl, 4.7; glucose, 11.1; MgSO_4_, 1.2; KH_2_PO_4_, 1.2; CaCl, 2.5; 2-[[1,3-dihydroxy-2-(hydroxymethyl)propan-2-yl]amino]ethanesulfonic acid, 10; pH, 7.4). Primary myocytes were isolated by digestion in 2 mg/mL collagenase (Type IV, Fisher Scientific, Loughborough, United Kingdom) in Dulbecco’s modified Eagle’s medium for up to 1 h at 37°C. The cells were released by trituration through fire-polished glass pipettes. Freshly isolated myocytes were plated, prior to transformation and selection, in Dulbecco’s modified Eagle’s medium supplemented with 10% foetal calf serum, penicillin (100 IU/mL), and streptomycin (100 μg/mL).

Transformation: TEFLYA cells producing retroviruses either hTERT or a temperature-sensitive mutation of SV40 U19tsA58 Δ89-97 were cultured in DMEM supplemented with 10% FBS and antibiotics (penicillin/streptomycin plus 100 ug/mL hygromycin for TEFLYA hTERT and 1.5 mg/mL G418 for TEFLYA SV40 U19tsA58 Δ89-97) at 37°C and 5% CO_2_ (O'Hare et al., 2001). Virus-containing supernatants were harvested after growing near confluent cultures in a T75 flask for 12 h in a 10 mL growth medium without antibiotic selection. Supernatants from both virus-producing lines were filtered through a 0.45-µm filter and mixed 1:1, and this virus stock was used immediately for the transduction of primary cells.

The primary myometrial cells were cultured in Dulbecco’s modified Eagle medium (DMEM)/F12 supplemented with 10% foetal calf serum, penicillin (100 IU/mL), and streptomycin (100 μg/mL) in a T75 flask. For virus transduction, the medium was removed and replaced with 5 mL of virus stock plus 5 mL of fresh growth medium and 16 µL of 5 mg/mL polybrene (8 μg/mL final concentration). The medium was replaced the next day with 14 mL of fresh growth medium and allowed to grow for 3 days, passaging into a new flask as required. Four days after transduction, the cells were seeded at low density into 14-cm Petri dishes, and 0.25 μg/mL G418 and 30 μg/mL hygromycin B were added for selection of stable transformants. Three days after the start of the selection, the cells were cultured at a permissive temperature of 33°C to activate SV40 large T antigen. Individual colonies were picked using cloning discs and transferred into a 96-well plate after 2–4 weeks and expanded into 24-well and 6-well plates. Conditionally immortalised MYLA cells were maintained for rapid proliferation at 33°C and analysed at 37°C when the large T antigen was inactive. The cells were authenticated with ASN-002 short tandem repeat (STR) profiling by Eurofins Genomics Europe Applied Genomics GmbH (Supplementary Materials S3) to provide a reference for future maintenance of cell purity and genomic integrity. The cells were subcultured at 90% confluency by lifting with 0.05% trypsin and not used beyond passage 12.

### Literature FP antagonists

Literature FP antagonists were synthesised using the described literature methodologies or purchased from a commercial supplier. N582707 was synthesised by Ferring Research Institute Inc. (San Diego, CA, United States) using the described methodologies. All compounds were solvated in DMSO to 10 mM and then diluted prior to use in biological assays. The chemical names and literature references (Martos et al., 2016; Pohl et al., 2018; Beck et al., 2020) are listed in Table 1.

### Synthesis of novel FP antagonist N582707

A complete schema for the synthesis of N582707 is shown in Figure 1. All reactions were carried out in an oven-dried round-bottomed flask under an inert nitrogen atmosphere with stirring. Solvents, reagents, and chemicals were purchased from various sources and used as received unless otherwise noted. Spectra for ^1^H were recorded at room temperature with a Bruker PA BBO 400S1 BBF-H-D-05 Z SP (400 MHz) spectrometer or a Varian ASW probe (400 MHz) unless otherwise noted. Chemical shifts are reported in δ (ppm) relative units to residual solvent peaks CDCl3 (7.26 ppm for ^1^H) and DMSO-d6 (2.50 ppm for ^1^H and 39.5 ppm for ^13^C). Splitting patterns are assigned as s (singlet), d (doublet), t (triplet), multiplet (m), and dd (doublet of doublet). Mass spectrometry measurements were recorded using the Vanquish Horizon UHPLC System connected to Thermo Orbitrap Q Exactive Plus in high-resolution positive mode. The predicted masses were extracted to ±5 ppm.

Tetrahydrothiophen-3-ol (**B**): To a mixture of **A** (20 g, 195.78 mmol, 16.67 mL, 1 eq) in THF (200 mL) was added BH_3_-THF (1 M, 195.78 mL, 1 eq) in one portion at 0°C under N_2_. The mixture was stirred at 20°C for 12 h. TLC (petroleum ether:ethyl acetate = 3:1, Rf = 0.40) showed that the reaction was complete. The residue was poured into MeOH (10 mL) and stirred for 30 min. The aqueous phase was extracted with EtOAc (3 × 10 mL). The combined organic phase was washed with brine (3 × 10 mL), dried with Na_2_SO_4_, filtered, and concentrated under vacuum. The residue was purified by silica flash column chromatography to afford 18.0 g (88% yield) of **B** as a white solid.

Tetrahydrothiophen-3-yl methanesulfonate (**C**): To a mixture of **B** (18 g, 172.79 mmol, 1 eq) in DCM (200 mL) was added DMAP (31.67 g, 259.19 mmol, 1.5 eq) in one portion at 0°C under N_2_. Then, MsCl (23.75 g, 207.35 mmol, 16.05 mL, 1.2 eq) was added. The mixture was stirred at 20°C for 12 h. TLC (petroleum ether:ethyl acetate = 1:1, Rf = 0.45) showed that the reaction was complete. The residue was poured into water (10 mL) and stirred for 3 min. The aqueous phase was extracted with DCM (3 × 10 mL). The combined organic phase was washed with brine (10 mL × 3), dried with Na_2_SO_4_, filtered, and concentrated under vacuum. The residue was purified by silica flash column chromatography to afford 29.5 g (93.7% yield) of **C** as a white solid.

Tetrahydrothiophene-3-carbonitrile (**D**): To a mixture of **C** (29.5 g, 161.86 mmol, 1 eq) in DMF (300 mL) was added sodium cyanide (39.66 g, 809.28 mmol, 5 eq) in one portion at 70°C under N_2_. The mixture was stirred at 70°C for 12 h. TLC (petroleum ether:ethyl acetate = 5:1, Rf = 0.35) showed that the reaction was complete. The residue was poured into water (10 mL) and stirred for 3 min. The aqueous phase was extracted with MTBE (3 × 20 mL). The combined organic phase was washed with brine (3 × 20 mL), dried with Na_2_SO_4_, filtered, and concentrated under vacuum. The residue was purified by silica flash column chromatography to afford 10.8 g (59% yield) of **D** as a white solid.

N-methoxy-N-methyltetrahydrothiophene-3-carboxamide (**E**): A solution of **D** (5.25 g, 46.39 mmol, 1 eq) in EtOH (10 mL) was added to a solution of NaOH (19.30 g, 482.42 mmol, 10.4 eq) in H_2_O (223.1 mL) and EtOH (112.9 mL). The mixture was stirred at 100°C for 3 h. TLC (petroleum ether: ethyl acetate = 5:1, Rf = 0) showed that the reaction was complete. The reaction was quenched with 1 M of hydrochloric acid to adjust pH to 3. The mixture was concentrated under vacuum. The mixture was dissolved in DCM (20 mL), dried with MgSO_4_, filtered, and concentrated under vacuum. The crude compound was used as is for the next step.

The crude compound and N-methoxymethanamine (4.53 g, 46.39 mmol, 1 eq) in DCM (60 mL) was added 50% T3P (35.42 g, 55.67 mmol, 33.1 mL, 1.2 eq) and TEA (14.1 g, 139.2 mmol, 19.37 mL, 3 eq) in one portion at 20°C under N_2_. The mixture was stirred at 20°C for 12 h. TLC (DCM: MeOH = 15:1, Rf = 0.7) showed that the reaction was complete. The residue was poured into water (10 mL) and stirred for 3 min. The aqueous phase was extracted with DCM (3 × 10 mL). The combined organic phase was washed with brine (3 × 10 mL), dried with Na_2_SO_4_, filtered, and concentrated under vacuum. The residue was purified by flash column chromatography (SiO_2_, DCM:MeOH = 15:1, Rf = 0.7) to afford 6.89 g (85% yield, two steps) of **E** as a white solid.

(4-Fluorophenyl)(tetrahydrothiophen-3-yl)methanone (**F**): To a mixture of **E** (6.89 g, 39.32 mmol, 1 eq) in THF (150 mL) was added (4-fluorophenyl)magnesium bromide (1 M, 157.26 mL, 4 eq) in one portion at 0°C under N_2_. The mixture was stirred at 0°C for 1 h. TLC (petroleum ether:ethyl acetate = 5:1, Rf = 0.52) showed that the reaction was complete. The residue was poured into NH_4_Cl (10 mL). The mixture was extracted with ethyl acetate (2 × 10 mL). The organic phase was washed with brine (10 mL), dried with Na_2_SO_4_, filtered, and concentrated under vacuum. The residue was purified by flash column chromatography (SiO_2_, petroleum ether:ethyl acetate = 5:1, Rf = 0.52) to afford 8.25 g (99.8% yield) of **F** as a white solid.

(1,1-Dioxidotetrahydrothiophen-3-yl)(4-fluorophenyl)methanone (**G**): To a mixture of **F** (10 g, 47.56 mmol, 1 eq) in DCM (50 mL) was added 80% m-CPBA (30.78 g, 142.68 mmol, 3 eq) in one portion at 0°C under N_2_. The mixture was stirred at 0°C for 1 h. TLC (petroleum ether: ethyl acetate = 1:1, Rf = 0.43) showed that the reaction was complete. The residue was poured into Na_2_SO_3_ (100 mL) and stirred for 3 min. The aqueous phase was extracted with DCM (20 mL). The combined organic phase was washed with saturated NaHCO_3_ (30 mL) and washed with brine, dried with Na_2_SO_4_, filtered, and concentrated under vacuum. The residue was purified by flash column chromatography (petroleum ether/ethyl acetate = 5/1 to 1/1) to afford 7.29 g (73% yield) of **G** as a white solid.

(*E*)-3-((4-fluorophenyl)(hydroxyimino)methyl)tetrahydrothiophene 1,1-dioxide (**H**): To a mixture of **G** (2.1 g, 8.67 mmol, 1 eq) in MeOH (83 mL) was added NaOAc (10.38 g, 126.56 mmol, 14.6 eq) and NH_2_OH∙HCl (1.20 g, 17.34 mmol, 2 eq) in one portion at 20°C under N_2_. The mixture was stirred for 12 h. TLC (petroleum ether:ethyl acetate = 1:1, Rf = 0.43) showed that the reaction was complete. The mixture was concentrated in vacuum. The residue was poured into water (50 mL) and stirred for 3 min. The aqueous phase was extracted with ethyl acetate (3 × 50 mL). The combined organic phase was washed with brine (3 × 50 mL), dried with anhydrous Na_2_SO_4_, filtered, and concentrated under vacuum. The residue was purified by flash column chromatography (SiO_2_, petroleum ether/ethyl acetate = 5/1 to 1/1) to afford 2.21 g of **H** as a white solid.

(2*S*)-N-((1,1-dioxidotetrahydrothiophen-3-yl)(4-fluorophenyl)methyl)-1-((4-(pyridin-3-yl)phenyl)sulfonyl)azetidine-2-carboxamide (**I**): Intermediate **H** (4.4 g, 17.10 mmol, 1 eq) was dissolved in MeOH (480 mL) and NH_3_∙H_2_O (80 mL). Pd/C (4.4 g, 8.16 mmol, 10% purity) was added in one portion at 50°C. The suspension was degassed under vacuum and purged with H_2_ several times. The mixture was stirred under H_2_ (50 psi) at 50°C for 12 h. TLC (petroleum ether:ethyl acetate = 1:1, Rf = 0) indicated that **H** was consumed completely. The mixture was filtered under Celite and concentrated under vacuum.

To a solution of (S)-1-((4-(pyridin-3-yl)phenyl)sulfonyl)azetidine-2-carboxylic acid (5.44 g, 17.10 mmol, 1 eq) in DCM (35 mL), HOBt (3.47 g, 25.65 mmol, 1.5 eq), EDCI (4.90 g, 25.65 mmol, 1.5 eq), and TEA (7.14 mL, 51.3 mmol, 3 eq) were added. The mixture was stirred at room temperature for 30 min and then added to the abovementioned crude mixture. The mixture was stirred at room temperature for 12 h. TLC (DCM:MeOH = 15:1, Rf = 0.33) indicated that the reaction was complete. The reaction mixture was quenched by the addition of H_2_O (30 mL). The residue was extracted with DCM (3 × 50 mL). The combined organic layers were washed with brine (2 × 20 mL), dried over Na_2_SO_4_, filtered, and concentrated under vacuum. The residue was purified by flash column chromatography (SiO_2_, petroleum ether/ethyl acetate = 1/1 to 0/1) to afford 6.35 g (68.3% yield, two steps) of **I** as a white solid.

**N582707**: Stereoisomeric mixture **I** was subjected to preparative SFC separation using a Cellulose-2 column from Phenomenex, Torrance, CA, USA (250 mm × 30 mm, 10 um) and [0.1% NH_3_H_2_O ETOH]; B%: 55%–55%, min, as the mobile phase. Four initial peaks were observed (**P1**–**P4**), with peak 1 (**P1**) being the eutomer. Isolation of **P1** yielded 1.29 g (20.3% yield) of FE-0208599. ^1^H NMR (400 MHz, DMSO-d6) δ ppm 9.01 (s, ^1^H) 8.78 (d, J = 8.82 Hz, ^1^H) 8.67 (d, J = 4.85 Hz, ^1^H) 8.21 (br d, J = 7.94 Hz, ^1^H) 8.06 (d, J = 8.38 Hz, ^2^H) 7.93–8.00 (m, ^2^H) 7.57 (dd, J = 7.83, 4.74 Hz, ^1^H) 7.44 (dd, J = 8.38, 5.51 Hz, ^2^H) 7.19 (t, J = 8.82 Hz, ^2^H) 4.93 (br t, J = 8.82 Hz, ^1^H) 4.32 (t, J = 8.16 Hz, ^1^H) 3.71–3.81 (m, ^1^H) 3.61 (q, J = 8.01 Hz, ^1^H) 3.31–3.33 (m, ^1^H) 3.16–3.27 (m, ^1^H) 2.98–3.11 (m, ^1^H) 2.84–2.93 (m, ^2^H) 2.05–2.25 (m, ^2^H) 1.77–1.90 (m, ^1^H) 1.69 (br d, J = 5.07 Hz, ^1^H). ^13^C NMR (151 MHz, DMSO) δ 168.00, 162.23, 160.62, 147.16, 145.79, 140.98, 137.55, 136.45, 134.82, 134.33, 129.28, 129.02, 128.13, 125.04, 115.27, 115.12, 62.24, 55.38, 54.20, 51.65, 48.29, 41.12, 25.77, 19.18. Expected [M + H] C_26_H_27_FN_3_O_5_S_2_ = 544.1372; found [M + H] = 544.1366. All structural analysis data of N582707 can be found in Supplementary Materials S4–S8.

### Agonist and antagonist Ca^2+^ assay

MYLA cells were grown to 95%–100% confluency in 3 cm^2^ glass-bottom dishes (MatTek Corporation, MA, United States). The cells were serum starved for 24–48 h with 2% DMEM/F12 media and then incubated with 5 μM Calbryte^TM^ 520 AM (Stratech Scientific Ltd., Ely, United Kingdom) for 60 min at 37°C in a 95%/5% air/CO_2_-humidified environment, followed by 15 min at room temperature in the dark. The cells were washed and incubated in 2 mL modified Krebs–Henseleit (m-KHB) solution (composition (mM): NaCl, 133; KCl, 4.7; glucose, 11.1; MgSO_4_, 1.2; KH_2_PO_4_, 1.2; N-Tris (hydroxymethyl)methyl-2-aminoethanesulfonic acid, 10; CaCl_2_∙2H_2_O, 2.5; pH 7.4). The plates were then loaded on the stage of an Olympus IX81 inverted microscope and visualised with a 10x objective lens. Temperatures were maintained at 37°C. Calbryte^TM^ 520 AM was excited at Ex/Em: 490/525 nm. The cells were imaged for 3 min to allow any initial LED-induced Ca^2+^ signalling to subside. For live experiments, cells were challenged for 3 min with a half-log incremental concentration of PGF_2α_ or FP antagonist (300 p.m.–10 µM) in the presence of 1 µM PGF_2α_. Fluorescence was captured at a rate of one frame per second for 3 min, with agonist/antagonist injections occurring after a 30-s basal period. Then, 1 μM PGF_2α_ or 100 nM oxytocin was added as a positive control, and 10 µM ionomycin (Sigma Aldrich, Poole, United Kingdom) provided a GPCR-independent positive control of Ca^2+^ release and determined the F_max_ for analysis calculations.

Data analysis: Videos were visualised, and data were analysed using ImageJ, the image analysis software. Changes in fluorescence and area under the curve (AUC) of Ca^2+^ oscillations were calculated using the equation: ((raw data − background) − minimum background)/averaged ionomycin F_max_, and then percentage-corrected to stimulated (1 µM PGF_2α_). The frequency of Ca^2+^ oscillations was calculated as the average number of oscillations over time in seconds (Hz). All data were visualised using the GraphPad Prism 9 software.

### [^35^S]-guanosine 5′-O-[gamma-thio] triphosphate binding

Tissue preparation: MYLA cells were grown to 95%–100% confluency in a T175 flask, lifted, and pelleted via standard cell culture techniques. The harvested cells were homogenised in ice-cold lysis buffer (composition (mM): HEPES, 20; EDTA, 1; MgCl_2_, 2; KCl, 10; DTT, 2; pH, 7.4) using a Coleman handheld homogeniser. The homogenates were cleared (1,000×*g*, 10 min, 4°C) and membranes were collected by centrifugation (16,100×*g*, 90 min, 4°C). The membranes were resuspended in freezing buffer [composition (mM): HEPES, 10; MgCl_2_, 1; DTT, 1; pH, 7.4], where the protein concentration was adjusted to 1.5 mg/mL and rapidly frozen in liquid nitrogen. The membranes were stored at −80°C until required.

Radioligand binding assay: 75 μg of membrane was added to 50 μL of assay buffer [composition (mM): HEPES, 10; MgCl_2_, 10; NaCl, 100; pH, 7.4] containing 1 nM [^35^S]-GTPγS (1,250 Ci/mmol) and 10 μM GDP, with or without agonist and antagonists as required, and incubated at 30°C for 2 min. Non-specific binding (NSB) was determined by the inclusion of 10 μM unlabelled GTPγS. Incubation was terminated by the addition of 900 μL of ice-cold assay buffer, and the samples were transferred to ice. The cell membrane was recovered from the reaction mixture by centrifugation (16,100×*g*, 6 min, 4°C), and the supernatant was removed by aspiration. Membrane pellets were solubilised by the addition of 50 μL of ice-cold solubilisation buffer [composition (mM): Tris-HCl, 100; NaCl, 200; EDTA, 1; IGEPAL CA 630, 1.25% (v/v); 0.2% (w/v) SDS; pH, 7.4] and vortex mixing. Once the protein was completely solubilised, an equal volume of solubilisation buffer without SDS was added. The solubilised protein was pre-cleared with rabbit serum (1:100 dilution) and 30 μL of Protein-G Sepharose beads (Invitrogen, Paisley, United Kingdom) (protein-G Sepharose bead suspension 30% v/v in TE buffer [composition (mM): Tris-HCl, 10; EDTA, 10; pH, 8.0)] for 60 min at 4°C. The Protein-G Sepharose beads and any insoluble material were collected by centrifugation (16,100×*g*, 6 min, 4°C), and then 100 μL of the supernatant was transferred to a fresh tube containing G-protein–specific antiserum (1:100 dilution of anti-Gα_q/11/14_ antibody (G-7), anti-Gα_i-1_ antibody (R4), or anti-Gα_s_/_olf_ antibody (A-5); Santa Cruz Biotechnology, Inc., Heidelberg, Germany). The samples were vortex mixed and rotated overnight at 4°C. Then, 70 μL of 30% Protein-G Sepharose beads were added to each sample tube and vortex mixed before incubation for 90 min at 4°C. The protein-G Sepharose beads were pelleted (16,100×*g*, 6 min, 4°C) and the supernatant was removed. The beads were washed and pelleted thrice with 500 μL of solubilisation buffer (less SDS) before re-suspension in Pico-Flour^TM^ scintillation cocktail (Perkin Elmer, Buckinghamshire, United Kingdom) where [^35^S]-GTPγS was determined by standard liquid scintillation counting methods.

Data analysis: Specific binding was determined by CPM values and expressed as a % increase over basal (unstimulated), and plotted using the GraphPad Prism 9 software.

### RNA extraction and purity assessment

The MYLA cells were seeded into a T75 flask and allowed to grow to 95%–100% confluency. The cells were serum starved for 24 h and then treated with either 1 µM PGF_2α,_ PGF_2α_ vehicle (ethanol) equivalent or 1 µM PGF_2α_ + 1 µM FP antagonist at time (T) 0. The cells were harvested at T = 1, 3, 6, 9, 12, 24, and 48 h, pelleted (1,200 rpm, 5 min, 4°C), and snap frozen on dry ice before storage at −80°C.

RNA was extracted from pelleted cells using the GenElute^TM^ Total RNA Purification Kit (Sigma Aldrich, Poole, United Kingdom) according to the manufacturer’s instructions. RNA concentration and purity were determined using a NanoDrop 1000 Spectrophotometer (Thermo Fisher Scientific, Loughborough, United Kingdom). All RNA samples were stored at −80°C until use.

### cDNA generation and RT-qPCR

cDNA was generated using the VILO cDNA Synthesis Kit (Invitrogen, Paisley, United Kingdom) according to the manufacturer’s instructions and using the Veriti^TM^ 96-Well Fast Thermal Cycler (Thermo Fisher, Loughborough, United Kingdom).

RT-qPCR samples were technical triplicates of biological triplicates in 364-well optical plates. Amplification was performed in 10 μL reactions containing 5 μL of 2X EXPRESS qPCR SuperMix with premixed ROX reference dye (Thermo Fisher, Loughborough, United Kingdom), 0.5 μL of each specific TaqMan primer pair-probe (listed in Supplementary Materials S9), and 1 μL of cDNA or water control. qRT-PCR was performed using the Applied Biosystems QuantStudio 5 Real-Time PCR System (qPCR) with an initial denaturation for 10 min at 95 °C, primer annealing at 50 °C for 2 min, followed by 40 cycles of 15 s at 95 °C and 1 min at 60 °C.

Data analysis: The relative expression of the target genes was calculated using the delta-CT method as described by Pfaffl (2001), normalised to the geometric mean of three housekeeping genes (GAPDH, ACTB, and RPL19), and then plotted using the GraphPad Prism 9 software.

### RNA quality assessment, library preparation, and sequencing

RNA quality was verified using a Bioanalyzer according to the manufacturer’s instructions (Eukaryote Total RNA Nano, Agilent). Illumina TruSeq RNA libraries were prepared and sequenced using a NextSeq 500 with a high-output 75 bp cycle cartridge (Illumina, Cambridge, United Kingdom) by the University of Warwick Genomic Facility.

### RNA-sequencing bioinformatics analysis

bcl2fastq v2.20.0.422 was used for demultiplexing each sample. FastQC v0.11.9 was used to quality control check the demultiplexed FastQC files, and MultiQC v1.9 consolidated the QC reports. Reads were aligned to the human reference genome build GRCh38 release 86 using STAR version 2.7.9a. The number of reads mapped to each genomic feature was calculated with featureCounts v2.0.1. The counts were imported into R Studio and analysed with the DESeq2 package.

Data analysis: TPM data were processed and analysed using R Studio and then plotted using the GraphPad 9 software.

### Statistical analysis

#### Agonist and antagonist Ca^2+^ assay

Experiments were repeated on MYLA cells, where N represents the number of biological repeats. As provided in Figure 2 and Supplementary Materials S2, the data were analysed by one-way ANOVA with *post hoc* Dunnett’s multiple comparison test comparing agonist treatment to basal or antagonist treatment to 1 µM PGF_2α_-stimulated cells. The data calculated as *p* < 0.05 were considered statistically significant and are graphically represented as ****p < 0.0001, ***p < 0.001, **p < 0.01, and *p < 0.05. In Figure 4, data were analysed by two-way ANOVA with *post hoc* Dunnett’s multiple comparison test comparing antagonist treatment to vehicle-stimulated cells. Data calculated as *p* < 0.05 were considered statistically significant and are represented as ****p < 0.0001, ***p < 0.001, **p < 0.01, and *p < 0.05. As provided in Table 2 and Table 3, EC_50_, pEC_50_, IC_50_, pIC_50_, E_max_, and Hill slope values were determined by removing outliers using Grubb’s (alpha = 0.05) and then plotted using [log (agonist/inhibitor) vs. response–variable response (four parameters)] and constraining the bottom to 0 using the GraphPad Prism 9 software.

#### [^35^S]-guanosine 5′-O-[gamma-thio] triphosphate binding

The experiments were repeated on MYLA cell membranes, where N represents the number of biological repeats. The data in Figure 3 were analysed by one-tailed, unpaired t-tests comparing vehicle + 1 µM PGF_2α_ to basal and vehicle + 1 µM PGF_2α_ to 1 µM compound 39/40 + 1 µM PGF_2α._ Data calculated as *p* < 0.05 were considered significant and are graphically represented as **p < 0.01. Not significant results are depicted as ns.

#### RNA sequencing

The experiments were technical triplicates of biological triplicate samples taken from seven time points. The data in Figures 8–10 were analysed by two-way ANOVA and *post hoc* Dunnett’s multiple comparison test comparing treatments to the ethanol vehicle. Data calculated as *p* < 0.05 were considered statistically significant and are graphically represented as ****p < 0.0001, ***p < 0.001, **p < 0.01, and *p < 0.05.

#### RT-qPCR

The experiments were technical triplicates of biological triplicate samples taken from seven time points. The data in Figure 12 were analysed by one-way ANOVA and *post hoc* Dunnett’s multiple comparison test comparing treatments to 1 µM PGF_2α._ Data calculated as *p* < 0.05 were considered statistically significant and are graphically represented as ****p < 0.0001, ***p < 0.001, **p < 0.01, and *p < 0.05.

## Data Availability

The data presented in the study are deposited in the Gene Expression Omnibus repository, accession number GSE249529.
