# Supplementary material for: PGF2α induces a pro-labour phenotypical switch in human myometrial cells that can be inhibited with PGF2α receptor antagonists
Source: Front Pharmacol. 2023 Dec 14;14:1285779. doi: 10.3389/fphar.2023.1285779 (PMC10752971; doi:10.3389/fphar.2023.1285779)
Supplement: Supplementary file 1 [file Table1.DOCX]

Supplementary Materials

**PGF_2α_ induces a pro-labour phenotypical switch in human myometrial cells that can be inhibited with PGF_2α_ receptor antagonists**

Isabel Hamshaw^1^, Anne Straube^2^, Richard Stark^3^, Laura Baxter^3^, Mohammad T. Alam^3,4^, Walter J. Wever^5^, Jun Yin^5^, Yong Yue^5^, Philippe Pinton^5,6^, Aritro Sen^5^, Gregory D. Ferguson^5^, and Andrew M. Blanks^1,2*^

**^1^**Clinical Science Research Laboratories, Division of Biomedical Sciences, Warwick Medical School, University of Warwick, Coventry, UK.

**^2^**Centre for Mechanochemical Cell Biology & Division of Biomedical Sciences, University of Warwick, Coventry, UK.

**^3^**Bioinformatics RTP, Warwick Medical School, University of Warwick, Coventry, UK.

**^4^**Department of Biology, College of Science, United Arab Emirates University, Abu Dhabi, UAE.

^5^Ferring Research Institute Inc., San Diego, CA, USA.

^6^Ferring Pharmaceuticals, International PharmaScience Center, Kastrup, Denmark.

*Correspondence: Andrew M. Blanks, [andrew.blanks@warwick.ac.uk](mailto:andrew.blanks@warwick.ac.uk)

**Supplementary Materials 1: Effect of the vehicle for PGF_2α,_ DMSO, upon calcium transients in MYLA cells**. (A) Representative traces showing calcium transients in MYLA cells in response to the equivalent DMSO vehicle for PGF_2α_ plus a positive control of 100 nM oxytocin. Data are mean ± SD, N=3.

| **Supplementary Materials 2: Statistical significance of the effect of PGF_2α_, its vehicle and 100 nM oxytocin upon Ca2+ transience in MYLA cells as depicted in Figure 1.** Data are mean ± SD, N=3 (data were analysed by one-way ANOVA and Dunnett’s multiple comparison test comparing PGF2α and oxytocin treatment to basal. ns, not significant, **p* < 0.05, ***p* < 0.01, ****p* < 0.001 and *****p* < 0.0001. | | | |
| --- | --- | --- | --- |
| **Concentration** | **Effect on F_max_** | **Effect on AUC** | **Effect on frequency** |
| **300 pM PGF_2α_** | 0.9996 ns | 0.9997 ns | 0.6402 ns |
| **1 nM PGF_2α_** | 0.9996 ns | 0.9997 ns | 0.3064 ns |
| **3 nM PGF_2α_** | 0.9918 ns | 0.9994 ns | 0.1760 ns |
| **10 nM PGF_2α_** | 0.7048 ns | 0.9885 ns | 0.0024 ** |
| **30 nM PGF_2α_** | 0.1859 ns | 0.7669 ns | <0.0001 **** |
| **100 nM PGF_2α_** | 0.0008 *** | 0.1357 ns | <0.0001 **** |
| **300 nM PGF_2α_** | <0.0001 **** | 0.0521 ns | <0.0001 **** |
| **1 µM PGF_2α_** | <0.0001 **** | 0.0291 * | <0.0001 **** |
| **3 µM PGF_2α_** | <0.0001 **** | 0.0160 * | 0.0003 *** |
| **10 µM PGF_2α_** | 0.0006 *** | 0.1590 ns | 0.0053 ** |
| **300 pM vehicle equivalent** | 0.9997 ns | >0.9999 ns | >0.9999 ns |
| **1 nM vehicle equivalent** | 0.9994 ns | >0.9999 ns | >0.9999 ns |
| **3 nM vehicle equivalent** | 0.9996 ns | >0.9999 ns | >0.9999 ns |
| **10 nM vehicle equivalent** | 0.9997 ns | >0.9999 ns | >0.9999 ns |
| **30 nM vehicle equivalent** | 0.9999 ns | >0.9999 ns | >0.9999 ns |
| **100 nM vehicle equivalent** | 0.9992 ns | >0.9999 ns | >0.9999 ns |
| **300 nM vehicle equivalent** | 0.9999 ns | >0.9999 ns | >0.9999 ns |
| **1 µM vehicle equivalent** | >0.9999 ns | >0.9999 ns | >0.9999 ns |
| **3 µM vehicle equivalent** | 0.9998 ns | >0.999 ns | >0.999 ns |
| **10 µM vehicle equivalent** | 0.7426 ns | >0.9999 ns | >0.9999 ns |
| **100 nM oxytocin** | <0.0001 **** | <0.0001 **** | <0.0001 **** |


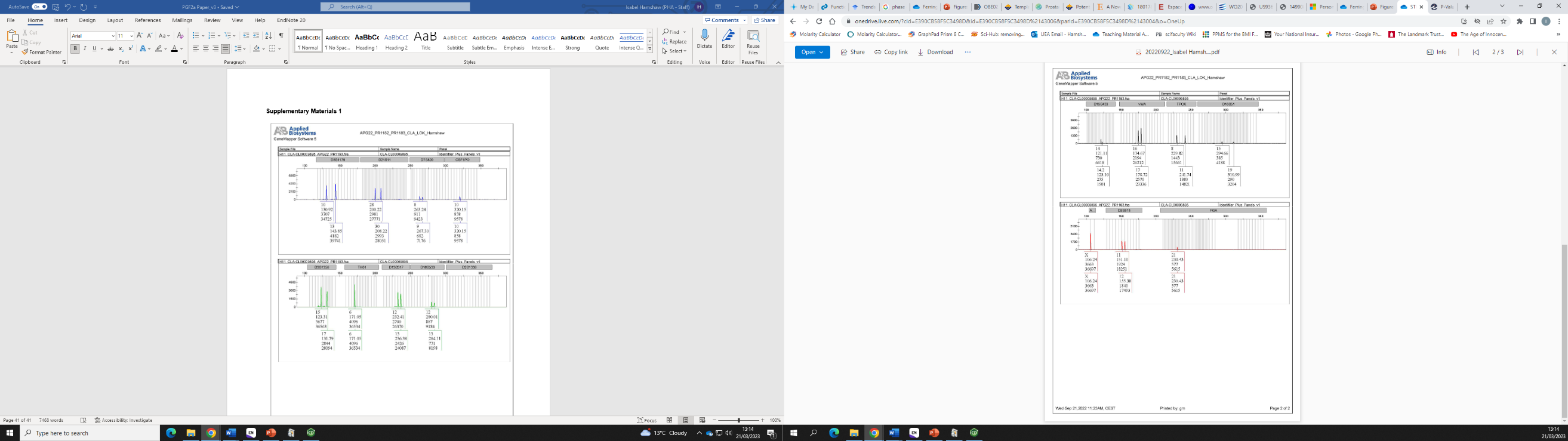

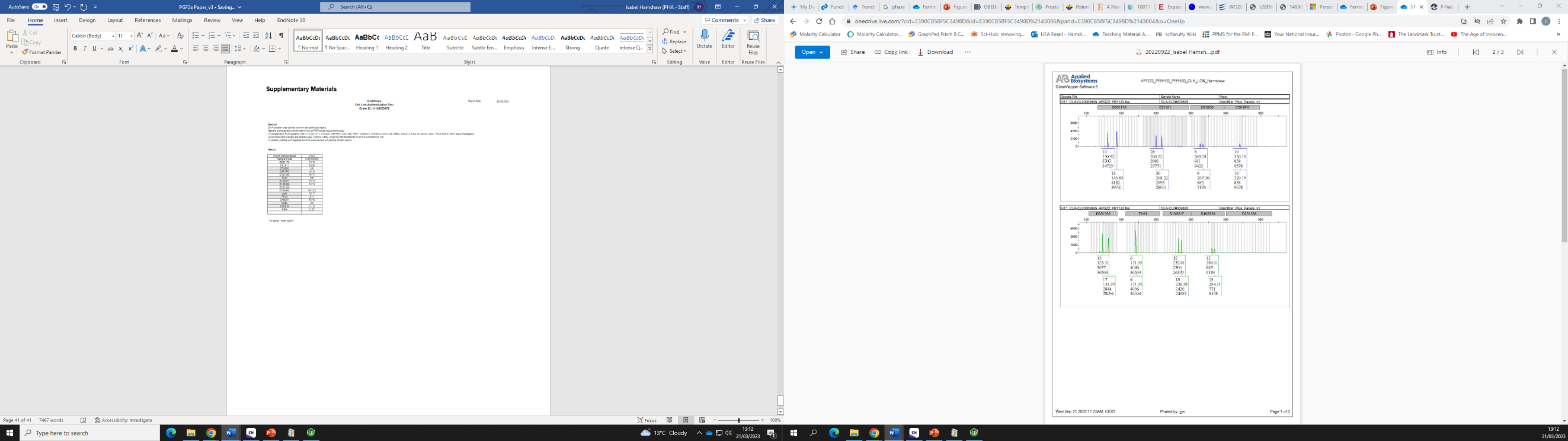
**Supplementary Materials 3: ASN-002 short tandem repeat (STR) profiling of MYLA cells.** MYLA cells were authenticated with ASN-002 short tandem repeat (STR) profiling by Eurofins Genomics Europe Applied Genomics GmbH.

**
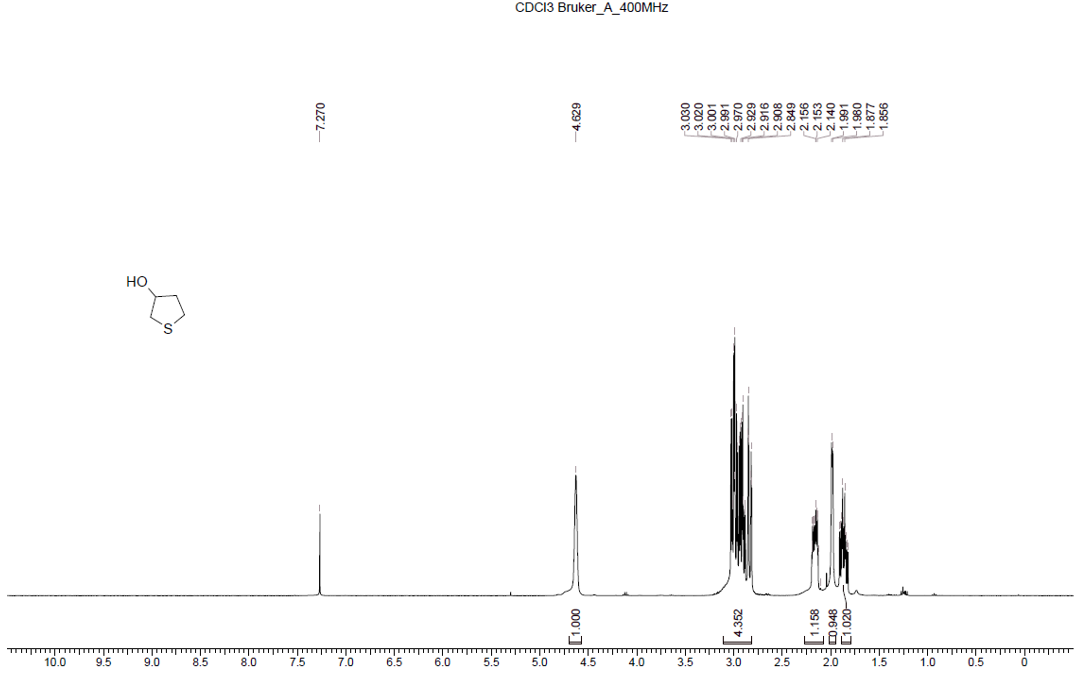
**

**
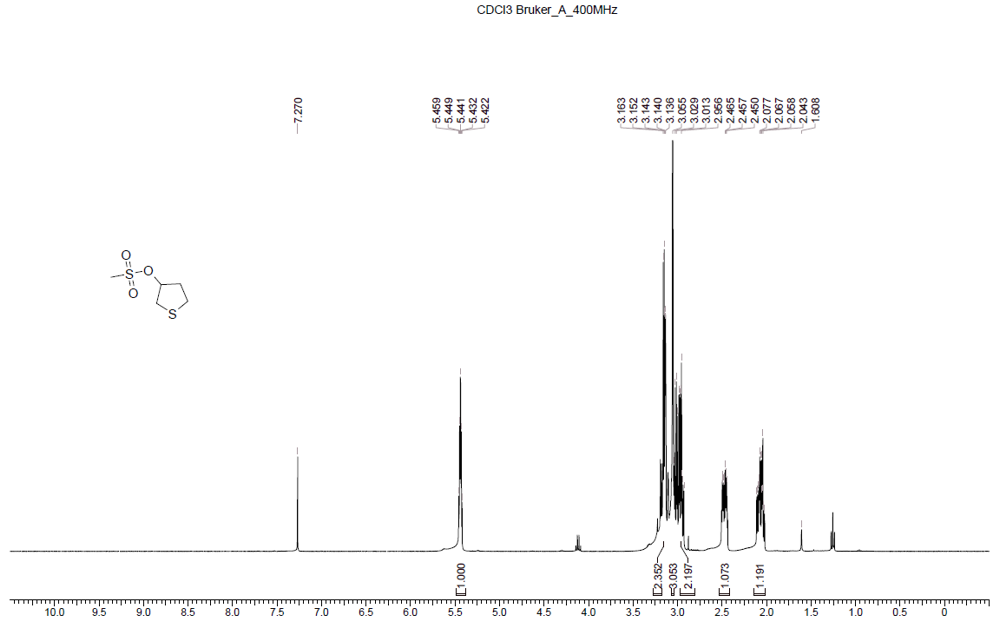
**

**
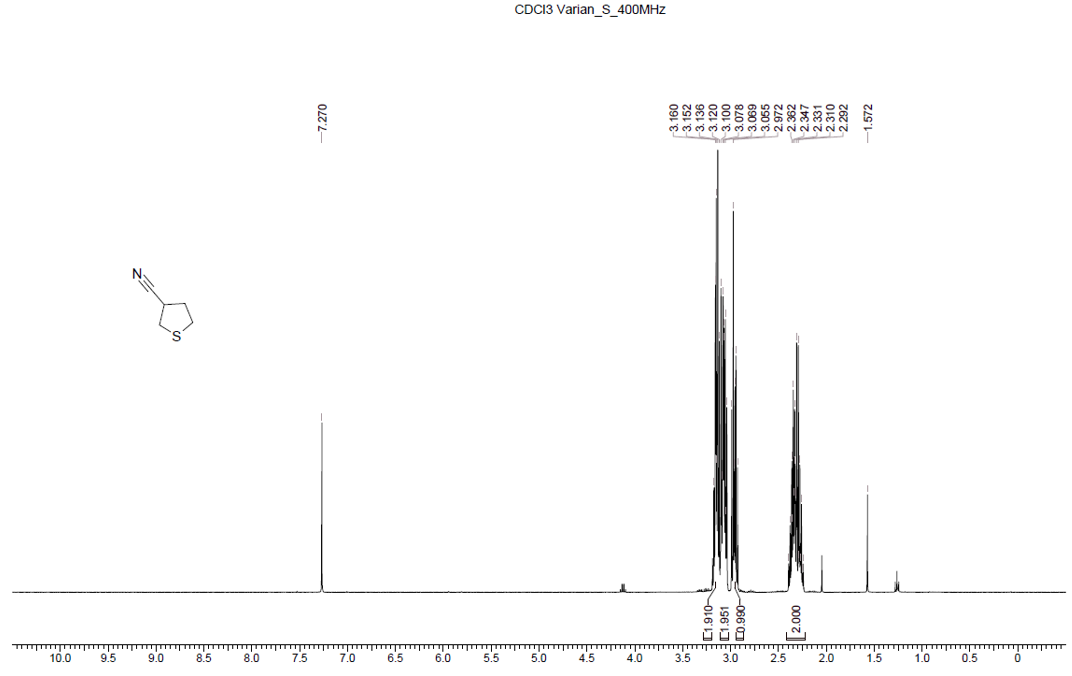
**

**
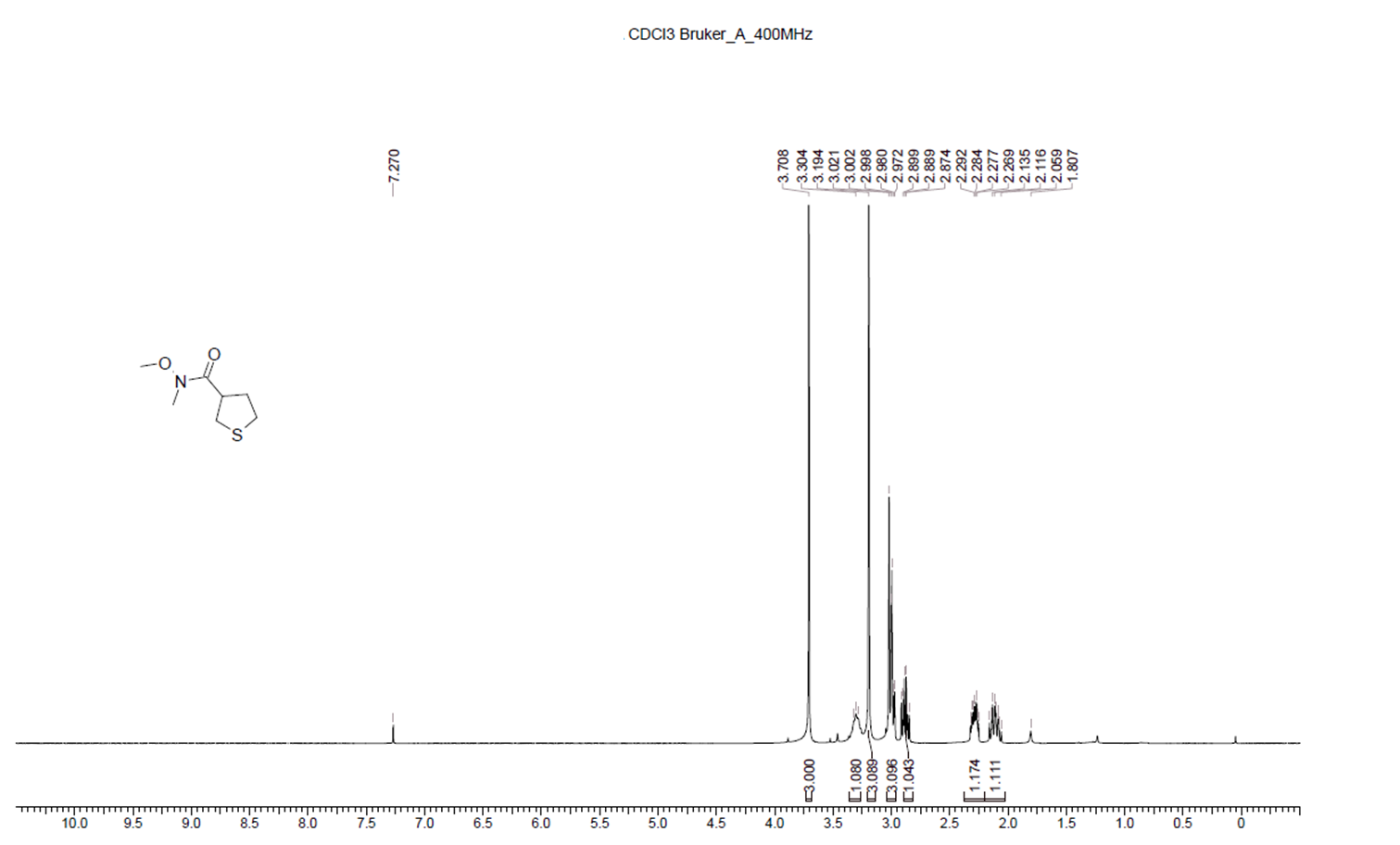
**

**
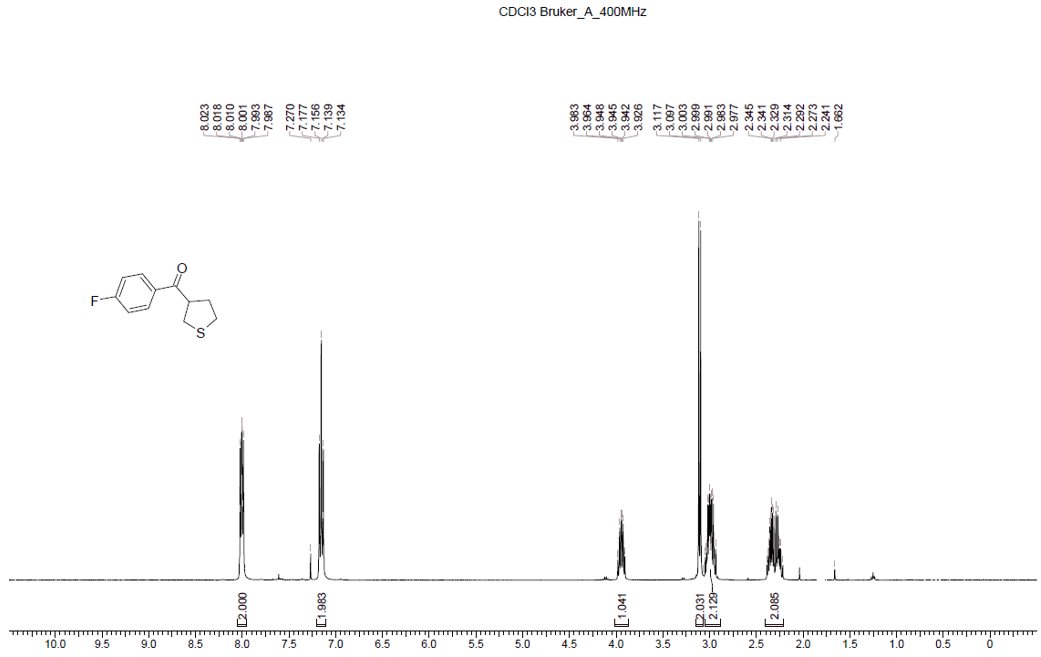
**

**
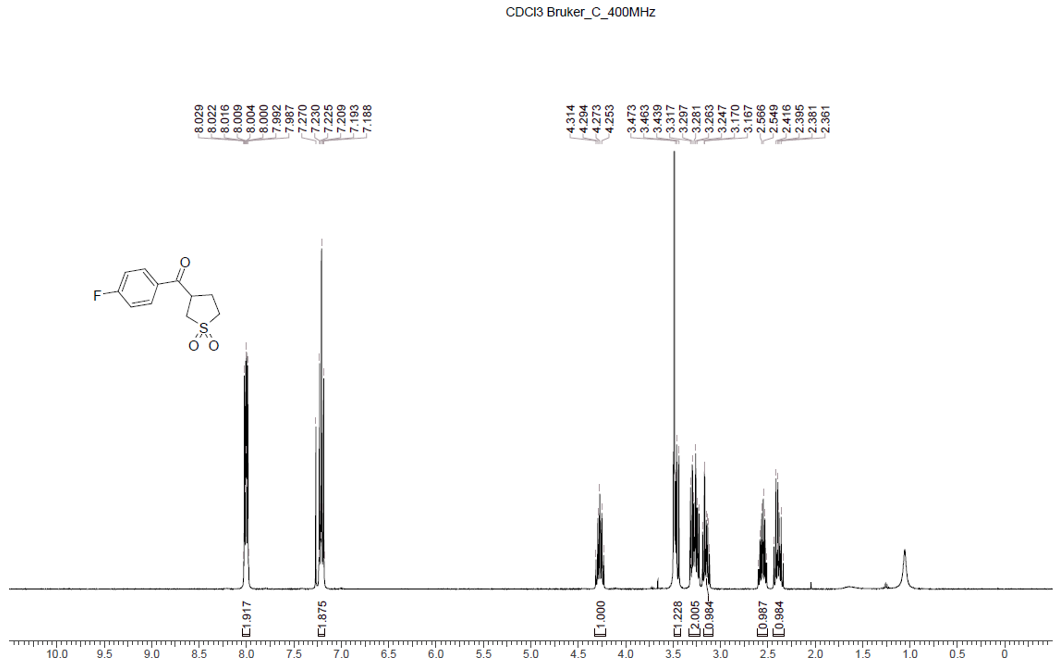
**

**
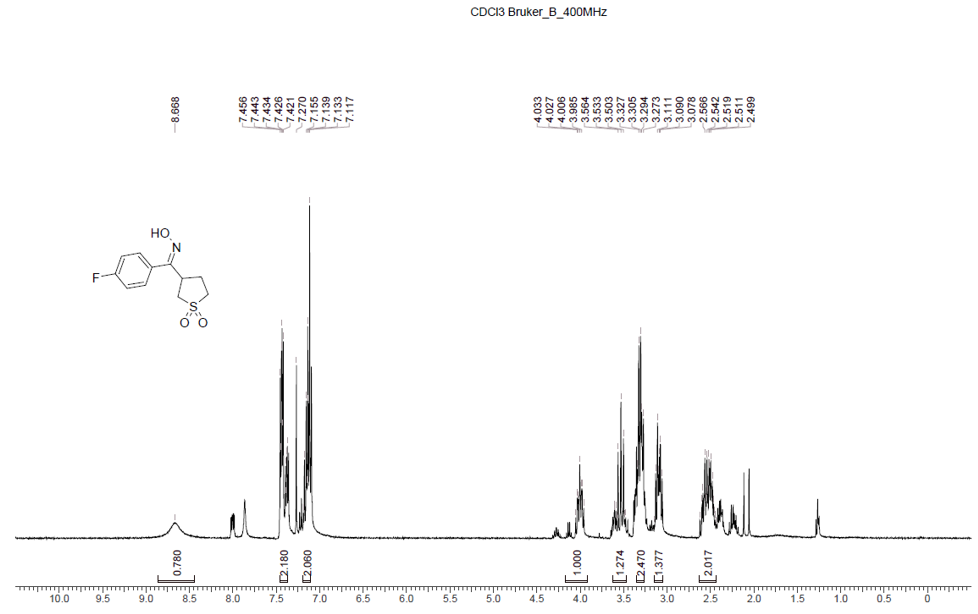
**


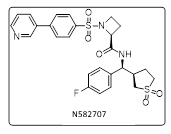
**
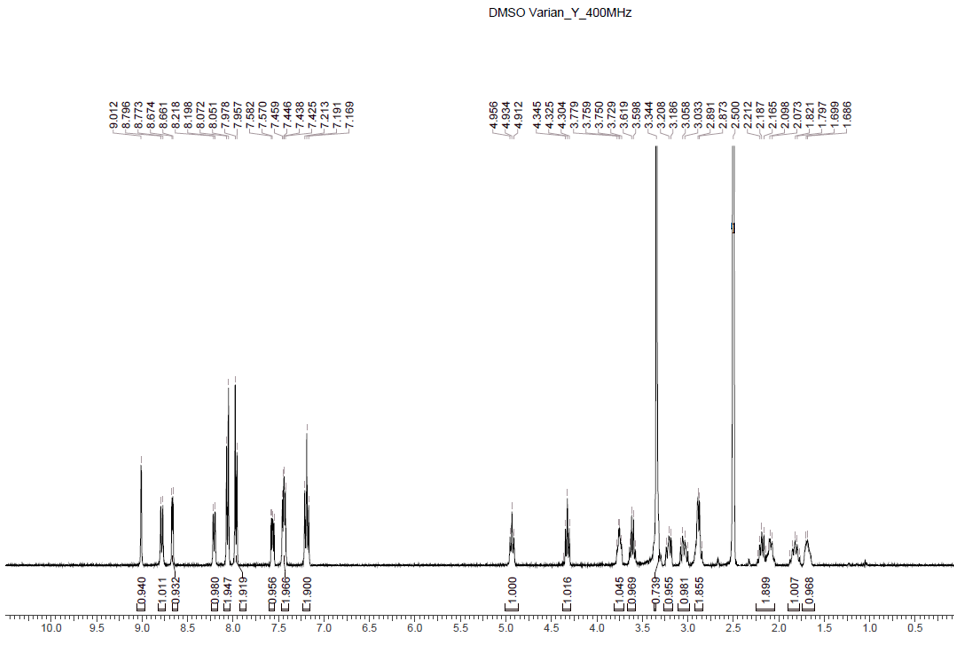
**


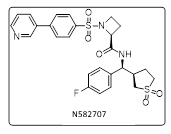


**Supplemental Materials 4: N582707 structural analysis and nuclear magnetic resonance (NMR).**

**
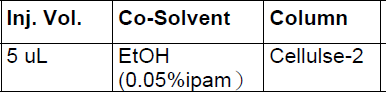

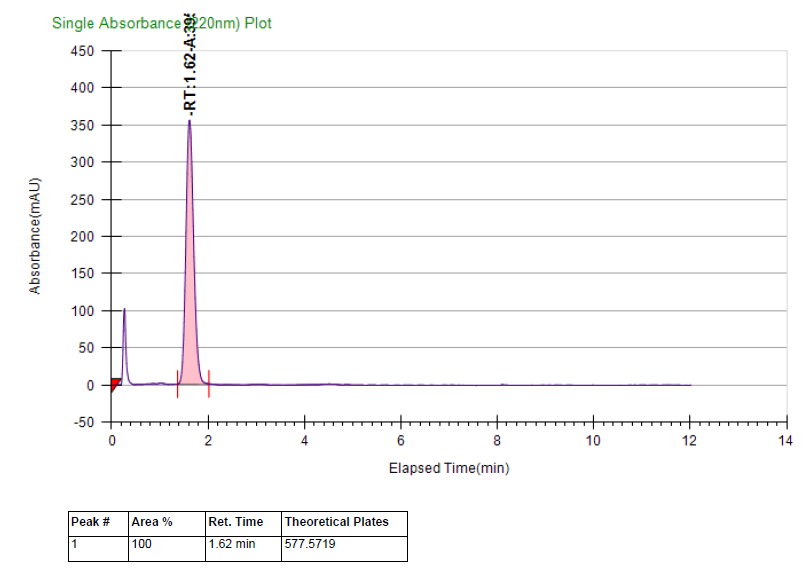
Supplemental Materials 5: Analytical supercritical fluid chromatography (SFC) of isolated N582707.**

**
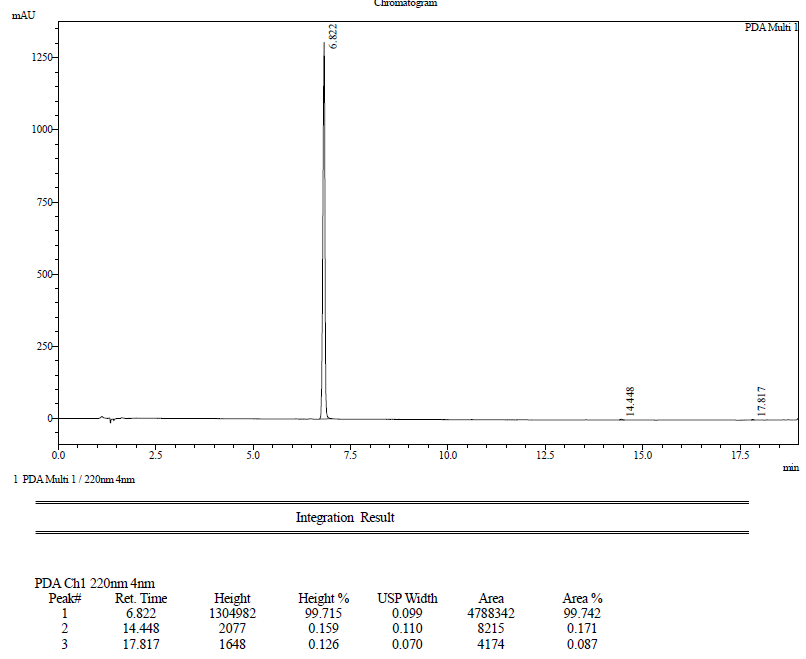
**

**Supplementary Materials 6: Analytical high-performance liquid chromatography (HPLC) of isolated N582707.**

**
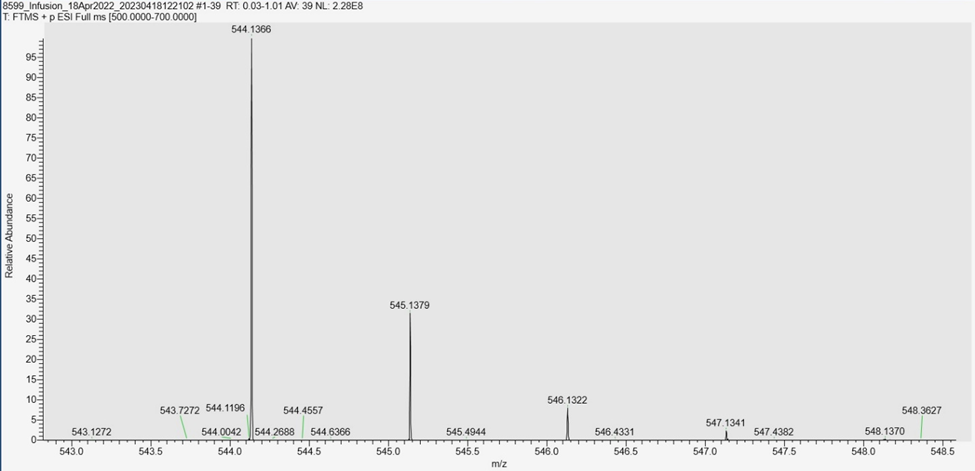
Supplementary Materials 7: High resolution mass spectrometry (HRMS) of isolated N582707.**

**
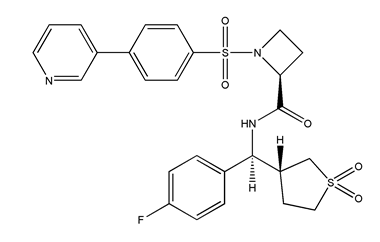
**

**Supplementary Materials 8: Absolute configuration determination by crystal structure analysis of N582707.** A crystal with size of 0.06×0.14×0.18mm was used for X-ray diffraction data collection. The data were collected on a Bruker D8 Venture Photon II area-detector diffractometer using CuKα radiation by φ/w scan mode. 20358 reflections were collected, of which 4896 reflections were unique ((I/sigma≥2) = 3919). The structure was solved by direct methods and all of the non-H atoms were refined by full-matrix least-squares methods. R1=0.0393, wR2=0.1250(w=1/s|F|2), S=1.063. The final stoichiometric formula is C26H26FN3O5S2. Calculated molecular weight is 543.62. Calculated crystal density is 1.411g/cm3.

| **Supplementary Materials 9: List of RT-qPCR TaqMan probes.​** | | |
| --- | --- | --- |
| **Target**​ | **ID Number**​ | **Base pair/kDa**​ |
| ACTB​ | Hs01060665_g1​ | 83​ |
| GAPDH​ | Hs02758991_g1​ | 93​ |
| RPL19​ | Hs02338565_gH​ | 127​ |
| CALD1​ | Hs00921987_m1​ | 69​ |
| MYLK​ | Hs00364926_m1​ | 112​ |
| OXTR​ | Hs00168573_m1​ | 84​ |

**Supplementary Materials 10. Expression of cell line selection markers.**

MYLA cell line mRNA (TPM, mean ± standard deviation) expression of selection markers for myometrial smooth muscle. Expression of OXTR (pregnant myometrial smooth muscle) and CALD1 (smooth muscle) was high, whilst expression of AVPR1A (high is vascular smooth muscle), ALDH1A1 (high in fibroblasts) was low and expression of CD90 (high in fibroblasts) was below the limit of detection.
